# Correction: Population genetic analysis of *Aedes aegypti* reveals evidence of emerging admixture populations in coastal Kenya

**DOI:** 10.1371/journal.pntd.0013631

**Published:** 2025-10-22

**Authors:** Francis Mulwa, Dario Balcazar, Solomon Langat, James Mutisya, Betty Chelangat, Carolyn S. McBride, Noah Rose, Jeffrey Powell, Rosemary Sang, Armanda Bastos, Andrea Gloria-Soria, Joel Lutomiah

There are errors in the author affiliations. The correct affiliations are as follows:

Francis Mulwa1,2, Dario Balcazar3, Solomon Langat1, James Mutisya1, Betty Chelangat1, Carolyn S. McBride4, Noah Rose4, Jeffrey Powell5, Rosemary Sang1, Armanda Bastos2,6, Andrea Gloria-Soria3, Joel Lutomiah1

**1** Center for Virus Research, Kenya Medical Research Institute, Nairobi, Kenya, **2** Department of Zoology & Entomology, University of Pretoria, Pretoria, South Africa, **3** Department of Entomology, The Connecticut Agricultural Experiment Station, New Haven, Connecticut, United States of America, **4** Department of Ecology and Evolutionary Biology, University of Princeton, Princeton, New Jersey, United States of America, **5** Ecology and Evolutionary Biology Department, Yale University, New Haven, Connecticut, United States of America, **6** Department of Veterinary Tropical Diseases, University of Pretoria, Pretoria, South Africa.
