# Supplementary material for: Population genetic analysis of Aedes aegypti reveals evidence of emerging admixture populations in coastal Kenya
Source: PLoS Negl Trop Dis. 2025 May 20;19(5):e0013041. doi: 10.1371/journal.pntd.0013041 (PMC12140423; doi:10.1371/journal.pntd.0013041)
Supplement: S1 Table — (DOCX) [file pntd.0013041.s001.docx]

**SI_Table: Kenyan *Aedes aegypti* populations.**

| **County** | **Area** | **Site** | **Sample abbreviation** | **Species** | **Sex** | **Number Analysed** | **Year collected** | **Coordinates** |
| --- | --- | --- | --- | --- | --- | --- | --- | --- |
| Busia | Malaba | Railway station | GN/001 | Ae. aegypti | F | 1 | 2022 | N00.63166 E034.26680 |
| Busia | Malaba | Railway station | GN/002 | Ae. aegypti | F | 1 | 2022 | N00.63166 E034.26680 |
| Busia | Malaba | Railway station | GN/003 | Ae. aegypti | F | 1 | 2022 | N00.63166 E034.26680 |
| Busia | Malaba | Railway station | GN/004 | Ae. aegypti | F | 1 | 2022 | N00.63166 E034.26680 |
| Busia | Malaba | Railway station | GN/005 | Ae. aegypti | F | 1 | 2022 | N00.63166 E034.26680 |
| Busia | Malaba | Railway station | GN/006 | Ae. aegypti | F | 1 | 2022 | N00.63166 E034.26680 |
| Busia | Malaba | Railway station | GN/008 | Ae. aegypti | F | 1 | 2022 | N00.63166 E034.26680 |
| Busia | Malaba | Immigration HQ | GN/009 | Ae. aegypti | F | 1 | 2022 | N00.63527 E034.27346 |
| Busia | Malaba | Immigration HQ | GN/011 | Ae. aegypti | F | 1 | 2022 | N00.63527 E034.27346 |
| Busia | Malaba | Immigration Quaters | GN/012 | Ae. aegypti | F | 1 | 2022 | N00.63527 E034.27346 |
| Busia | Malaba | Immigrtion HQ | GN/013 | Ae. aegypti | F | 1 | 2022 | N00.63527 E034.27346 |
| Busia | Malaba | Immigration HQ | GN/014 | Ae. aegypti | F | 1 | 2022 | N00.63527 E034.27346 |
| Kisumu | Kisumu | Railway Station | GN/031 | Ae. aegypti | F | 1 | 2022 | S00.10745 E034.74898 |
| Kisumu | Kisumu | Railway Station | GN/032 | Ae. aegypti | F | 1 | 2022 | S00.10745 E034.74898 |
| Kisumu | Kisumu | Railway Station | GN/033 | Ae. aegypti | F | 1 | 2022 | S00.10745 E034.74898 |
| Kisumu | Kisumu | Railway Station Marine School | GN/034 | Ae. aegypti | F | 1 | 2022 | S00.10745 E034.74898 |
| Kisumu | Kisumu | Railway Station Marine School | GN/035 | Ae. aegypti | F | 1 | 2022 | S00.10745 E034.74898 |
| Kisumu | Kisumu | Railway Station | GN/036 | Ae. aegypti | F | 1 | 2022 | S00.10745 E034.74898 |
| Kisumu | Kisumu | Railway Station Marine School | GN/037 | Ae. aegypti | F | 1 | 2022 | S00.10745 E034.74898 |
| Kisumu | Kisumu | Railway Station Marine School | GN/039 | Ae. aegypti | F | 1 | 2022 | S00.10745 E034.74898 |
| Kisumu | Kisumu | Railway Station Marine School | GN/040 | Ae. aegypti | F | 1 | 2022 | S00.10745 E034.74898 |
| Kisumu | Kisumu | Railway Station Querters | GN/041 | Ae. aegypti | F | 1 | 2022 | S00.10671 E034.74959 |
| Kisumu | Kisumu | Railway Station Querters | GN/042 | Ae. aegypti | F | 1 | 2022 | S00.10671 E034.74959 |
| Kisumu | Kisumu | Railway Station Querters | GN/043 | Ae. aegypti | F | 1 | 2022 | S00.10671 E034.74959 |
| Nakuru | Nakuru | Lenana Primary School | GN/061 | Ae. aegypti | F | 1 | 2022 | S00.28250 E036.07885 |
| Nakuru | Nakuru | Lenana Primary School | GN/062 | Ae. aegypti | F | 1 | 2022 | S00.28250 E036.07885 |
| Nakuru | Nakuru | Lenana Primary School | GN/063 | Ae. aegypti | F | 1 | 2022 | S00.28250 E036.07885 |
| Nakuru | Nakuru | Lenana Primary School | GN/064 | Ae. aegypti | F | 1 | 2022 | S00.28250 E036.07885 |
| Nakuru | Nakuru | Railway Station Querters | GN/065 | Ae. aegypti | F | 1 | 2022 | S00.28250 E036.07885 |
| Nakuru | Nakuru | Railway Station Querters | GN/066 | Ae. aegypti | F | 1 | 2022 | S00.28250 E036.07885 |
| Nakuru | Nakuru | Railway Station Querters | GN/067 | Ae. aegypti | F | 1 | 2022 | S00.28250 E036.07885 |
| Nakuru | Nakuru | Lenana Primary School | GN/068 | Ae. aegypti | F | 1 | 2022 | S00.28250 E036.07885 |
| Nakuru | Nakuru | Lenana Primary School | GN/069 | Ae. aegypti | F | 1 | 2022 | S00.28250 E036.07885 |
| Nakuru | Nakuru | Menengai High School | GN/070 | Ae. aegypti | F | 1 | 2022 | S00.28633 E036.07787 |
| Nakuru | Nakuru | Menengai High School | GN/071 | Ae. aegypti | F | 1 | 2022 | S00.28633 E036.07787 |
| Uasin Gishu | Eldoret | Maili Nne | GN/093 | Ae. aegypti | F | 1 | 2022 | N00.57243 E035.23129 |
| Uasin Gishu | Eldoret | Maili Nne | GN/095 | Ae. aegypti | F | 1 | 2022 | N00.57229 E035.23103 |
| Uasin Gishu | Eldoret | Maili Nne | GN/098 | Ae. aegypti | F | 1 | 2022 | N00.57229 E035.23103 |
| Uasin Gishu | Eldoret | Maili Nne | GN/099 | Ae. aegypti | F | 1 | 2022 | N00.57243 E035.23129 |
| Uasin Gishu | Eldoret | Maili Nne | GN/100 | Ae. aegypti | F | 1 | 2022 | N00.57243 E035.23129 |
| Uasin Gishu | Eldoret | Maili Nne | GN/101 | Ae. aegypti | F | 1 | 2022 | N00.57243 E035.23129 |
| Uasin Gishu | Eldoret | Pioneer | GN/105 | Ae. aegypti | F | 1 | 2022 | N00.51228 E035.27496 |
| Uasin Gishu | Eldoret | Pioneer | GN/106 | Ae. aegypti | F | 1 | 2022 | N00.51228 E035.27496 |
| Uasin Gishu | Eldoret | Eldoret Junction | GN/109 | Ae. aegypti | F | 1 | 2022 | N00.55310 E035.31150 |
| Uasin Gishu | Eldoret | Eldoret Junction | GN/111 | Ae. aegypti | F | 1 | 2022 | N00.55310 E035.31150 |
| Mombasa | Mvita | Railway station | GN/136 | Ae. aegypti | F | 1 | 2021 | S04.05725 E039.66151 |
| Mombasa | Mvita | Railway station | GN/137 | Ae. aegypti | F | 1 | 2021 | S04.05725 E039.66151 |
| Mombasa | Mvita | Railway station | GN/138 | Ae. aegypti | F | 1 | 2021 | S04.05725 E039.66151 |
| Mombasa | Mvita | Railway station | GN/139 | Ae. aegypti | F | 1 | 2021 | S04.05725 E039.66151 |
| Mombasa | Mvita | Mama Ngina | GN/140 | Ae. aegypti | F | 1 | 2021 | S04.07869 E039.67017 |
| Mombasa | Mvita | Mama Ngina | GN/141 | Ae. aegypti | F | 1 | 2021 | S04.07869 E039.67017 |
| Mombasa | Mvita | Mama Ngina | GN/142 | Ae. aegypti | F | 1 | 2021 | S04.07869 E039.67017 |
| Mombasa | Mvita | Railway station | GN/144 | Ae. aegypti | F | 1 | 2021 | S04.05725 E039.66151 |
| Mombasa | Mvita | Railway station | GN/145 | Ae. aegypti | F | 1 | 2021 | S04.05725 E039.66151 |
| Mombasa | Mvita | Kenya Ports Authority (KPA) | GN/146 | Ae. aegypti | F | 1 | 2021 | S04.06075 E039.65479 |
| Mombasa | Mvita | Kenya Ports Authority (KPA) | GN/147 | Ae. aegypti | F | 1 | 2021 | S04.06075 E039.65479 |
| Kilifi | Mariakani | Railway station | GN/158 | Ae. aegypti | F | 1 | 2021 | S03.85781 E039.46391 |
| Kilifi | Mariakani | Railway station | GN/159 | Ae. aegypti | F | 1 | 2021 | S03.85781 E039.46391 |
| Kilifi | Mariakani | Majengo | GN/161 | Ae. aegypti | F | 1 | 2021 | S03.86923 E039.47507 |
| Kilifi | Mariakani | Majengo | GN/162 | Ae. aegypti | F | 1 | 2021 | S03.86923 E039.47507 |
| Kilifi | Mariakani | Majengo | GN/163 | Ae. aegypti | F | 1 | 2021 | S03.86923 E039.47507 |
| Kilifi | Mariakani | Majengo | GN/164 | Ae. aegypti | F | 1 | 2021 | S03.86923 E039.47507 |
| Kilifi | Mariakani | Railway station | GN/165 | Ae. aegypti | F | 1 | 2021 | S03.85781 E039.46391 |
| Kilifi | Mariakani | Railway station | GN/166 | Ae. aegypti | F | 1 | 2021 | S03.85781 E039.46391 |
| Kilifi | Mariakani | Railway station | GN/167 | Ae. aegypti | F | 1 | 2021 | S03.85781 E039.46391 |
| Kilifi | Mariakani | Railway station | GN/168 | Ae. aegypti | F | 1 | 2021 | S03.85781 E039.46391 |
| Kilifi | Mariakani | Railway station | GN/169 | Ae. aegypti | F | 1 | 2021 | S03.85781 E039.46391 |
