# Supplementary material for: Population genetic analysis of Aedes aegypti reveals evidence of emerging admixture populations in coastal Kenya
Source: PLoS Negl Trop Dis. 2025 May 20;19(5):e0013041. doi: 10.1371/journal.pntd.0013041 (PMC12140423; doi:10.1371/journal.pntd.0013041)
Supplement: S3 Table — (DOCX) [file pntd.0013041.s003.docx]

|  | Mariakani | Malaba | Nakuru | Eldoret | Mombasa | Kisumu |
| --- | --- | --- | --- | --- | --- | --- |
| Mariakani | 0 | 0.001 | 0.001 | 0.001 | 0.001 | 0.001 |
| Malaba | 0.001 | 0 | 0.001 | 0.004 | 0.001 | 0.001 |
| Nakuru | 0.001 | 0.001 | 0 | 0.001 | 0.001 | 0.001 |
| Eldoret | 0.001 | 0.004 | 0.001 | 0 | 0.001 | 0.001 |
| Mombasa | 0.001 | 0.001 | 0.001 | 0.001 | 0 | 0.001 |
| Kisumu | 0.001 | 0.001 | 0.001 | 0.001 | 0.001 | 0 |
