# Supplementary material for: Population genetic analysis of Aedes aegypti reveals evidence of emerging admixture populations in coastal Kenya
Source: PLoS Negl Trop Dis. 2025 May 20;19(5):e0013041. doi: 10.1371/journal.pntd.0013041 (PMC12140423; doi:10.1371/journal.pntd.0013041)
Supplement: Attachment — Submitted filename: PNTD-D-24-01782 _Review recommendations_A....pdf [file pntd.0013041.s004.pdf]

PLOS Neglected Tropical Diseases

Population genetic analysis of Aedes *aegypti* reveals evidence of emerging hybrid Populations in coastal Kenya
----Manuscript Draft Review--

**PNTD-D-24-01782 Review recommendations**

This is to acknowledge that this is an original and important research paper addressing the Population genetic analysis of the two devastating subspecies of *Aedes aegypti*, implicated in the transmission of dengue and chikungunya virus in Kenya. The results generated could pave the way in the understanding the distribution of epidemics and facilitate the species management and the control of the disease. But has minor and majors comments:

**Minor corrections**

Line 150: How did the author screened SNPs using chip, in order to meet the Mendelian expectations.

Line 152: the author should avoid vague expression such as …to eliminate SNPs .which did not genotype well, instead ,use technical and appropriate expressions such as … to eliminate missing data at those specific locations on the genome .Or simply state that … to eliminate missing data which did not confidently allow to identify the genetic variation at those SNP sites.

The statistical data analysis methods section is missing in this paper

**Major corrections**

Line 109. Methods. The author should include a section on data quality control/check

Line 147- 148. The author should describe clarify the call rate data and present data analysis on an additional file to allow data replication and reproducibility.

Line 150-.151 the Mendelian expectations based on SNPs screening, please clarify.

Line 155. Should include data quality check, this full section is missing.

Line 160-170: The sample genotype calls rates are produced by the Axiom algorithm, but the results are not been presented on an additional file. The population stratification was done using PCA , to account for identification of the population structure at sample level. But it could have been better to perform a cross-validation technique in order to assess the robustness of results.

Line 170-171. At SNPs level, data quality control is described using the PLINK 1.9, to identify and remove conflicting SNPs that deviate significantly from expected patterns. Describe briefly the method used for quality check and validation at SNPs level. This will include, Minor Allele Frequency (MAF), access the SNPs Linkage disequilibrium (LD) and the Hardy-Weinberg equilibrium (HWE).

Line 178. Only Heterozygosity rate is described for genetic diversity but PIC test procedures if included could have been an asset.

.Results

Line 200: Before presenting Population structure results, the author should start first, data quality check and validation results. An additional file should be attached showing quality check outcome at sample-level, and at the SNPs level.

Line 229. Only the population stratification results using PCA are shown, presenting the population structure at sample level. Please, provide evidence of data validation at sample and SNPs level.

All genetic Diversity indices should be presented in a table

**Discussion**

Discuss first all SNPs validation and genetic diversity Indices before structure and isolation by distance

**Recommendations**

This paper is addressing the Population genetic analysis of the two devastating subspecies of *Aedes aegypti*, implicated in the transmission of dengue and chikungunya virus in Kenya. Results generated could account for the understanding of the distribution of epidemics and envisage a better management of the species and the disease control, but:

1. The author should include a clear quality check and validation methods steps , then present related results in the results section
2. Section of genetic diversity indices results should be presented in a table form, to preside the structure, PCA and phylogenetic results.

The results presented in this paper, although important are insufficient and therefore, make the decision of the paper difficult. The methodology is not rigorous and could not clearly allow the reproducibility of the experiment. Since the results could not be confirmed and validated at the sample and at the SNPs level. This paper; although important, is unfit for publication in Plos Neglected Tropical Diseases at its current state and is therefore rejected.
