# Supplementary material for: Population genetic analysis of Aedes aegypti reveals evidence of emerging admixture populations in coastal Kenya
Source: PLoS Negl Trop Dis. 2025 May 20;19(5):e0013041. doi: 10.1371/journal.pntd.0013041 (PMC12140423; doi:10.1371/journal.pntd.0013041)
Supplement: Attachment — Submitted filename: PNTD-D-24-01782_R1_reviewer_WAO.pdf [file pntd.0013041.s006.pdf]

PLOS Neglected Tropical Diseases

Population genetic analysis of Aedes aegypti reveals evidence of emergingAdmixture populations in coastal Kenya

--Manuscript Draft--

**Manuscript Number:**	PNTD-D-24-01782R1
**Full Title:**	Population genetic analysis of Aedes aegypti reveals evidence of emergingAdmixture populations in coastal Kenya
**Short Title:**	Aedes aegypti genetic structure analysis in Kenya
**Article Type:**	Research Article
**Keywords:**	Aedes aegypti, admixture , population structure, genetic diversity, Single Nucleotide Polymorphism, Kenya
**Abstract:**	Background:<br>The Aedes aegypti mosquito is widespread in tropical and subtropical regions. There are two recognized subspecies; the invasive Aedes aegypti aegypti (Aaa) and the ancestral Aedes aegypti formosus (Aaf). Aaf is common throughout Kenya whereas Aaa, which was historically confined to coastal regions, has undergone a range expansion. In areas of sympatry, gene flow may lead to admixed populations with potential differences in vectorial capacity. We hypothesize that coastal Ae. aegypti populations have a higher proportion of Aaa ancestry than those from inland locations of Kenya, influenced by their distance to the coast.<br>Methodology<br>Adult Ae. aegypti mosquitoes were collected using Biogent (BG) sentinel traps baited with carbon-dioxide (CO_2) from cities and towns along the Kenyan northern transport corridor. Aedes aegypti population structure, genetic diversity, and isolation by distance were analyzed using genome-wide single nucleotide polymorphism (SNPs) datasets generated with an Ae. aegypti microarray chip targeting ≈50,000 SNPs. Kenyan Aedes aegypti populations were placed into a global context within a phylogenetic tree, by combining the Kenyan dataset with a previously published global database database.<br>Results<br>A total of a total of 67 Ae. aegypti mosquitoes population from Kenya were genotyped, we found that western Kenya Ae. aegypti constitute a genetically homogenous population that clusters with African Aaf, whereas coastal mosquitoes showed evidence of admixture between the two subspecies. There was a positive correlation (Observation = 0.869, p = 0.0023) between genetic distance (FST) and geographic distance, suggesting isolation by distance. The phylogenetic analysis and the genetic structure analysis suggest that an Asian Aaa population is the source of Aaa invasion into Kenya.<br>Conclusions<br>These results provide evidence of the emerging an admixed population of Ae. aegypti in coastal Kenya between the sylvatic Aaf and the domesticated-human preferring Aaa. The observed gene flow from Aaa into Kenya may positively influence Ae. aegypti vectorial capacity, potentially increasing human feeding preference, biting rates and vector competence and could be promoting the observed dengue and chikungunya outbreaks.
**Additional Information:**	
**Question**	**Response**
**Government Employee**<br><br>Are you or any of the contributing authors an employee of the United States government?	Yes - One or more authors are employees of the U.S. government.

Manuscripts authored by one or more US Government employees are not copyrighted, but are licensed under a CC0 Public Domain Dedication, which allows unlimited distribution and reuse of the article for any lawful purpose. This is a legal requirement for US Government employees.<br><br>This will be typeset if the manuscript is accepted for publication.	
**Financial Disclosure**<br><br>Enter a financial disclosure statement that describes the sources of funding for the work included in this submission. Review the submission guidelines for detailed requirements. View published research articles from *PLOS Neglected Tropical Diseases* for specific examples.<br><br>This statement is required for submission and **will appear in the published article** if the submission is accepted. Please make sure it is accurate.<br><br>**Funded studies**<br>Enter a statement with the following details:<br>• Initials of the authors who received each award<br>• Grant numbers awarded to each author<br>• The full name of each funder<br>• URL of each funder website<br>• Did the sponsors or funders play any role in the study design, data collection and analysis, decision to publish, or preparation of the manuscript?<br><br>Did you receive funding for this work?	The author(s) received no specific funding for this work.
**Competing Interests**<br><br>On behalf of all authors, disclose any competing interests that could be perceived to bias this work.	The authors have declared that no competing interests exist

This statement will be typeset if the manuscript is accepted for publication.<br><br>Review the instructions link below and PLOS NTDs' competing interests policy to determine what information must be disclosed at submission.	
**Data Availability**<br><br>Provide a **Data Availability Statement** in the box below. This statement should detail where the data used in this submission can be accessed. This statement will be typeset if the manuscript is accepted for publication.<br><br>Before publication, authors are required to make all data underlying their findings fully available, without restriction. Review our PLOS Data Policy page for detailed information on this policy. Instructions for writing your Data Availability statement can be accessed via the Instructions link below.	The datasets generated and analysed in this study are available in the supplementary material and in the manuscript. The data will also be available in the public repository

Manuscript
Click here to access/download;Manuscript;Manuscript_Aedes aegypti population genetics.docx

Population genetic analysis of *Aedes aegypti* reveals evidence of emerging Admixture populations in coastal Kenya

Francis Mulwa[1, 2], Dario Balcazar[3], Solomon Langat[1], James Mutisya[1], Betty Chelangat[1], Carolyn. S. McBride[4], Noah Rose[4], Jeffrey Powell[5], Rosemary Sang[1], Armanda Bastos[2, 6], Andrea Gloria-Soria[3], Joel Lutomiah[1]

[1]Center for Virus Research, Kenya Medical Research Institute, Nairobi, Kenya;

[2]Department of Zoology & Entomology, University of Pretoria, South Africa

[3] Department of Entomology. The Connecticut Agricultural Experiment Station, USA

[4]University of Princeton, USA

[5]Ecology and Evolutionary Biology Department. Yale University, USA

[6]Department of Veterinary Tropical Diseases, University of Pretoria, South Africa

Abstract

Background:

The *Aedes aegypti* mosquito is widespread in tropical and subtropical regions. There are two recognized subspecies; the invasive *Aedes aegypti aegypti* (*Aaa*) and the ancestral *Aedes aegypti formosus* (*Aaf*). *Aaf* is common throughout Kenya whereas *Aaa*, which was historically confined to coastal regions, has undergone a range expansion. In areas of sympatry, gene flow may lead to admixed populations with potential differences in vectorial capacity. We hypothesize that coastal *Ae. aegypti* populations have a higher proportion of *Aaa* ancestry than those from inland locations of Kenya, influenced by their distance to the coast.

Methodology

Adult *Ae. aegypti* mosquitoes were collected using Biogent (BG) sentinel traps baited with carbon-dioxide (CO_2) from cities and towns along the Kenyan northern transport corridor. *Aedes aegypti* population structure, genetic diversity, and isolation by distance were analyzed using genome-wide single nucleotide polymorphism (SNPs) datasets generated with an *Ae. aegypti* microarray chip targeting ≈50,000 SNPs. Kenyan *Aedes aegypti* populations were placed into a global context within a phylogenetic tree, by combining the Kenyan dataset with a previously published global database database.

Results

A total of a total of 67 *Ae. aegypti* mosquitoes population from Kenya were genotyped, we found that western Kenya *Ae. aegypti* constitute a genetically homogenous population that clusters with African *Aaf*, whereas coastal mosquitoes showed evidence of admixture between the two subspecies. There was a positive correlation (Observation = 0.869, p = 0.0023) between genetic distance (FST) and geographic distance, suggesting isolation by distance. The phylogenetic analysis and the genetic structure analysis suggest that an Asian Aaa population is the source of Aaa invasion into Kenya.

Conclusions

These results provide evidence of the emerging an admixed population of *Ae. aegypti* in coastal Kenya between the sylvatic Aaf and the domesticated-human preferring *Aaa*. The observed gene flow from *Aaa* into Kenya may positively influence *Ae. aegypti* vectorial capacity, potentially increasing human feeding preference, biting rates and vector competence and could be promoting the observed dengue and chikungunya outbreaks.

Key words: *Aedes aegypti*, admixture , population structure, genetic diversity, Single Nucleotide Polymorphism, Kenya

Author's summary

Aedes aegypti is of great public health concern due the viruses they transmit. The vector is highly invasive and is expanding to new geographic regions, quickly adaptiting to new environment. This study focuses ~~in~~ understanding the genetic structure of *Ae. aegypti* in cities along the northern transport corridor in Kenya, which are at risk of vector invasion. We analyzed *Ae. aegypti* populations using a panel of Single Nucleotide Polymorphism (SNPs) markers distributed across the genome. Our analysis show admixture in the coastal region between *Aaf* and *Aaa*, with Asian mosquitos being the putative source of *Aaa* ancestry, while the western populations are more related to African *Aaf*. This research provides a broad picture of the nature and dynamics of the *Ae. aegypti* populations across Kenya and sets the bases for further genetic studies focused on improving vector control strategies and developing novel mosquito control methods.

Introduction

Aedes aegypti is the primary vector for arboviruses such as dengue, chikungunya, Zika, and yellow fever viruses, which cause frequent outbreaks mostly in tropical and subtropical regions across the world (1). This species has two widely recognized subspecies: *Aedes aegypti formosus* (*Aaf*), the sylvatic type that dominates across most of sub-Saharan Africa, and *Aedes aegypti aegypti* (*Aaa*), the invasive type of notable epidemiological relevance due to its anthropophagic traits (2-5). *Aaa* is believed to have originated in the Sahel region of West Africa but has since successfully invaded the tropics around the world, and is increasingly reported in temperate regions where it is associated with emerging outbreaks of Zika, dengue and yellow fever (6). Human activities such

as unplanned urban expansion, population movement, and worldwide trade have contributed to the adaptation of this invasive species to the urban environment, and its association with native species (7-10). *Aedes*-borne arboviral infections have become a major public health concern in Kenya since 2004/2005 chikungunya outbreak, and are rapidly spreading to new geographic regions (11-14). Entomological investigations during risk assessment and routine surveillance activities have shown that *Ae. aegypti* is distributed across the country, but predominant in coastal Kenya (15-17).

Ae. aegypti micro geographic population structure in Africa has revealed the presence of admixed populations of the two subspecies (10, 18, 19). In Kenya, previous genetic analyses indicated that the *Ae. aegypti* found inside residential houses in the coastal region are descendants of the invasive subspecies *Aaa* introduced to the east coast of Kenya (18, 20). These foreign mosquitoes have coexisted with the native *Aaf* for at least 60 years and were last recorded in 2009 (21). The two forms share same habitat and breeding containers both indoors and outdoors and it is likely that interbreeding has occurred (22-24). A recent study reported that *Ae. aegypti* population in coastal Kenya is a hybrid of the two subspecies (22, 25). Thus the overlap of the two subspecies could lead to admixture, as observed in other parts of Africa (26, 27). As a result of gene flow from *Aaa* into Kenya, the resulting ecotype may exhibit enhanced vectorial capacity, such as high human attack rates and increased vector competence which could increase transmission of diseases (25, 28). Although some admixture between the two subspecies has been shown in Kenya (18), the extent of admixture across mainland Kenya is still largely unknown.

Genome-wide analysis using single nucleotide polymorphisms (SNPs), has proven powerful for high-resolution analysis of *Ae. aegypti* evolutionary history and invasion dynamics (22, 29, 30). Gaining a better understanding of the genetic structure of *Ae. aegypti* in Kenya, along with inferring *Aaa* invasion dynamics and levels of *Aaa* gene flow into the country, can provide valuable insights for controlling and forecasting future disease outbreaks. The Kenyan northern transport corridor is the main passageway connecting several countries in Eastern Africa. It is comprised by the meter gauge railway line and a highway that ferry passengers and cargo from Mombasa to neighboring border countries such as Uganda, Tanzania and South Sudan. Mombasa city is an international tourism hub, as well as a logistics and communications hub for neighboring countries, making it highly susceptible to invasion by foreign species. Initial invasion followed by human-mediated passive dispersal is likely to facilitate spread to other urban areas in mainland Kenya.

We performed population genetic analyses on *Ae. aegypti* collected from diverse ecological zones along a corridor from coastal Kenya to the mainland regions to better understand the *Aaa* admixture across the country. A SNP genotyping array which targets 50,000 SNPs (31) was employed to investigate the population structure of *Ae. aegypti* in Kenya and determine the genetic diversity and differentiation in the urban cities along the Kenyan northern transport corridor. Such knowledge allows inference of the invasion process and underpins modelling of the spread of disease and insecticide resistance. Additionally, it is useful for maintaining effective vector control strategies and provides insight into the epidemiology of arthropod-borne diseases such as dengue virus.

Methods

Ethical Approvals

Ethical approval for this study was obtained from Kenya Medical Research Institute's (KEMRI) Scientific and Ethics Review Unit (KEMRI/SERU/CVR/007-2023/4999)

Study site

Urban areas were selected in specific cities and towns along the Northern transport corridor in proximity to the railway stations and commercial routes (highways) where container trucks make overnight stopovers (Figure 1). These areas are at risk of importation of pathogens from epidemic areas in the coastal region and vector invasion via human-mediated passive transport. The urban areas included coastal Mombasa (Mama Ngina grounds and the Kenya Ports Authority –KPA– precincts) and Mariakani, and mainland Nakuru, Kisumu, Busia, Malaba and Eldoret City, designated as western regions. The four cities (Mombasa, Nakuru, Eldoret and Kisumu) experience high traffic because of increased local and international travel. Mariakani and Malaba are layover sites for cargo trains and road trucks.

Figure 1: Map showing the entomological sampling sites in towns and cities along the Northern transport corridor in Kenya. The map was designed using ArcMap 10.2.2 with the ocean and lakes base layer derived from Natural Earth, a free GIS data source (naturalearthdata). The locations were collected using a GPS gadget (garmin etrex 20, https://buy.garmin.com/en-US/US/p/518046), and the county boundaries for Kenya derived from Africa Open data (https://africaopendata.org/dataset/kenya-counties-shapefile).

Mosquito collection and identification

Adult *Ae. aegypti* were collected using BG-Sentinel (BGS) traps (Biogent) baited with 500g CO_2 (dry ice) from different study sites and identified using published morphological keys (32-34). To

avoid analyzing siblings, mosquitoes were collected from multiple sites across the sampling area. Ten BG sentinel traps were set up (6:00AM -12:00PM) outdoors and in the peri-domestic area at different locations within the study sites per day, during the long rain season. Mosquitoes were retrieved from the traps twice daily, in the morning and evening, and transported to a temporary site laboratory. The collection bags containing the trapped mosquitoes were carefully packed into biohazard bags. The mosquitoes were anesthetized using triethylamine for five minutes. Once anesthetized, the *Ae. aegypti* mosquitoes were identified to species level based on morphological characteristics. One individual mosquito was genotyped per sampling site. The use of multiple sampling sites and traps were sufficient to minimize sampling of siblings.

Mosquito Genotyping

DNA extraction

Genomic DNA was extracted from 11-12 individual females' per population using Qiagen DNeasy blood/tissue kit as per the manufacturer's protocol with an additional step of adding 4ul of RNAase A to each sample to eliminate RNA. DNA was eluted to a final volume of 200 µl of 1/10X TE buffer. Samples were concentrated and normalized to 12ng/ul using Amicon Ultra 0.5uL - 30K centrifugal units prior to genotyping.

Single Nucleotide Polymorphisms (SNPs) genotyping

For population structure and genetic diversity analysis, we generated a SNP data set with populations of *Ae. aegypti* from throughout its global distribution, with a focus on the Kenyan population (summarized in supplementary Table S1). Genotyping was done with the *Ae. aegypti* Axiom_aegypti1 SNP chip (31) (Life Technologies Corporation CAT#550481)(35) at the University of North Carolina Functional Genomics Core, Chapel Hill. This genotyping array

contains 50,000 SNPs and was developed using representative Aedes aegypti populations worldwide to effectively capture the species' genetic diversity. It also enables the identification of *Ae. aegypti* subspecies (*Ae. aegypti aegypti* or *Ae. aegypti formosus*) (35, 36) and has been recently validated against whole-genome data (37). Additional data from previously described samples from populations of *Ae. aegypti* collected worldwide were used as a reference panel (S1 table 2). Data files were processed at Yale University with the Axiom Analysis Suite v.3.1. (Affymetrix, Santa Clara, CA) to retain those with high- quality samples (sample's dish quality control (DQC) ≥ 0.82, sample's QC call rate (QC) ≥ 66, Average call rate for passing samples (ACRPS) ≥ 90) and to call the genotypes. We genotyped 11-12 individuals *Ae. aegypti* mosquito per population at 50,000 single-nucleotide polymorphisms using the high-throughput genotyping chip, Axiom_aegypti1. The resulting dataset was complemented with previously described samples from several populations of *Ae. aegypti* collected worldwide (22, 38). From the Kenyan and global datasets, loci that fail to genotype at 80% or more and individuals with high levels of missing data were excluded of the individuals from the global dataset were filtered out from the loci obtained from the SNP-chip that met Mendelian expectations, using the – geno 0.2 option in plink 1.9 (39). Additionally, loci in linkage disequilibrium and those with a minor allele frequency (MAF) below 0.01 were removed (indep-pairwise 50 10 0.3, maf 0.01). Out of the 50,000 SNPs screened with the chip, 22,849 met Mendelian expectations according to Evans et al 2015 (35) and were retained. The SNP data were then filtered in PLINK v 1.9 (40, 41) to eliminate missing data at those specific locations on the genome that did not genotype well. We also removed variants that were missing in more than 20% of individuals by specifying flag --geno 0.2. Subsequently, individuals missing more than 5% of the remaining SNPs were removed with the –mind 0.05 option.

Admixture analyses

Geographic population structure was evaluated via Maximum Likelihood estimation of individual ancestries using the method implemented by the software Admixture 1.3.0 (42) which identifies genetic clusters and assigns proportions of each individual's ancestry to these clusters. The most likely number of clusters (K) was determined by conducting 10 independent runs from K = 1 to 10. We performed 10 independent runs for each K and results were summarized and plotted using the online version of CLUMPAK (43) and DISTRUCT v.1.1 (44). The optimal K was chosen by cross validating the Q values of the ten runs using the online software Structure Harvester. The Q value with the least logarithm was considered by looking at the least value on the scatter plot (45). To complement the admixture analysis, we performed principal component analysis (PCA) using the R ggplot2 package in R studio (46, 47). PCA is a statistical method of data visualization commonly used in population genetics to identify structure in the distribution of genetic variation across geographical locations. The result provides a framework for interpreting PCA projections in terms of underlying processes, including migration, geographical isolation, and admixture. For the PCA analysis SNP vector data file was performed using PLINK 1.9 (40) –pca command and plotted in R studio R Core (47).

Pearson correlation test

To assess whether *Aaa* ancestry increased with distance from the coast, *Aaa* ancestry proportions were plotted against each population distance from Mombasa and analyzed using a linear regression using R studio (44, 45) and the correlation coefficient was calculated. The genetic correlation between *Aaa* ancestry proportions—estimated using admixture at K = 2 from the global dataset—and the distance of each population from Mombasa was analyzed to assess *Aaa* introgression levels along a coastal-to-inland gradient, from Mombasa to western cities in

mainland Kenya. Linear correlation analysis was performed to assess the statistical relationship between *Aaa* ancestry and distance from Mombasa (Km) using Pearson's correlation coefficient test.

Genetic diversity

Average observed (Ho) and expected (He) heterozygosities were estimated from the SNPs dataset that included all populations from Kenya in GenoDive 3.04 (48, 49). Genetic diversity and differentiation, pairwise genetic distances (F_{ST}) between all pairs of populations and their significance (significance level of 0.05) were calculated in GenoDive, using 1,000 permutations. The Genepop file generated in PGDSpider (50) was used in GenoDive to determine pairwise differentiation and genetic diversity (51). The partitioning of the genomic variation among and within populations was evaluated through a hierarchical analysis of molecular variance, AMOVA (52), as implemented in Genodive, using 1,000 permutations. F_{ST} significance was tested using 999 permutations; standard deviations on F-statistics were obtained through bootstrapping over loci, and 95% confidence interval of F-statistics were obtained through bootstrapping over loci.

Isolation by distance

To assess the significance of the correlation between geographic and genetic distance matrices (isolation by distance; IBD), for all Kenyan populations, we performed a Mantel test with 999 permutations in "ade4" package (53) within R v.3.4.4 (46). The geographic distance matrix was produced from geographic coordinates in the Geographic distance matrix generator v. 1.2.3 (54). The correlation between geographic and genetic distance was plotted in R v.3.4.4 (46) and the correlation coefficient was calculated.

Phylogenetic analysis with the SNP data set

We evaluated the evolutionary relationships of Kenyan populations of *Ae. aegypti*, in the global context using maximum-likelihood inference on the population-level SNP data set with ascertainment bias correction for invariant sites, as implemented in IQ-Tree (55, 56). We allowed IQ-Tree to select the best-fitting substitution model, and assessed clade support using ultrafast bootstrap values.

Results

SNPs quality control and validation

After quality filtering, 22,849 of the 50,000 genotyped SNPs remained. Genotype calls were generated using Affymetrix Axiom Analysis Suite 3.1 (Life Technologies–Thermo Fisher Scientific) for both newly genotyped samples and previously studied populations. This software assigns genotypes based on probe fluorescence values while excluding probes (and their corresponding SNPs) with fluorescence deviations that may indicate probe misbinding. For probe quality control and SNP calling, we primarily used default software settings in Axiom Analysis Suite to filter out genotype calls from SNPs potentially affected by off-target variants, such as those arising from insertions, deletions, or mutations at probe sites.

Population structure

A total of 67 *Ae. aegypti* mosquitoes were genotyped; Mombasa (N=12), Mariakani (N=11), Nakuru (N=11), Eldoret (N=11), Kisumu (N=11), and Malaba (N=11) (for details on metadata see S1 Table1). These data were analyzed together with data available from previous studies used in other publications (22, 38). The Evanno method (57) identified run K=2 as the optimal number of clusters. However, clustering among six *Ae. aegypti* populations were best described by K = 3.

The individual admixture analysis on the Kenyan dataset (Figure 2 i, K = 2) showed that the populations belong to two genetic clusters. The first cluster included samples collected from various sites in the western region (Nakuru, Eldoret, Kisumu and Malaba). The second cluster primarily included samples from coastal region (Mombasa and Mariakani). At K=3, the data showed a third cluster consisting of samples collected from Nakuru and Eldoret. The *Ae. aegypti* populations from Mombasa in the coastal region showed a homogeneous genotype compared to other populations (Fig2 ii A) for the K=2.

Figure 2: Admixture bar plots for individual *Ae. aegypti* from Kenya (**Fig2i)**. Population names are reported on the *x*-axis. The *y*-axis reports the probability of assignment from each individual (*Q*-value) to one of the genetic groups identified, which are represented by different colors. Each vertical bar represents an individual. Individuals with 100% assignment to one group are identified by a single color. Bars with different percentages of colors represent individuals with mixed ancestry; the thick black lines within the plots indicate population limits. **Fig2ii**: Admixture bar plots for Kenyan *Ae. aegypti* population.

The Admixture analysis of *Ae. aegypti* at the global scale (summarized in supplementary 2 (S2), including historical samples (Fig. 3) indicate that there is significant admixture in *Ae. aegypti* populations from the coastal region (Fig. 3A K=2). The Evanno method (57) identified run K=2 as the optimal number of clusters. The coastal population displays admixture with *Aaa* populations. Focusing on the continental African dataset (Fig 3A, 3B), western Kenya populations cluster together with Gabon, Cameroon and Angola populations that are of the *Aaf* subspecies of *Ae. aegypti*.

Figure 3: Global Admixture bar plots for world *Ae. aegypti* population and Kenyan study population. Population names are reported on the x-axis. The y-axis reports the probability of assignment from each individual (Q-value) to one of the genetic groups identified, which are represented by different colors. Each vertical bar represents an individual. Individuals with 100% assignment to one group are identified by a single color. Bars with different percentages of colors represent individuals with mixed ancestry; the thick black lines within the plots indicate population limits.

The Kenyan *Ae. aegypti* populations form two genetically distinct clusters as observed in PCA, the coastal populations clusters on the right side of the plot, while Western population cluster to the left (figure4A). Consistent with global genome-wide SNP data, coastal populations overlap with the *Aaa* populations outside Africa, and are closer to Asian populations. PCA analysis of SNP genotypes on global data set (Fig.4B) shows that Kenyan populations are spread into three genetic clusters. One clusters with Cameroon and Gabon (Nakuru, Eldoret, Kisumu and Malaba), the other with South Africa (Mariakani), and the third one with Asia (Mombasa and Mariakani) (Figure 4B). The PCA confirms the results obtained from Admixture analysis. The result indicate that Kenya is not a pure *Aaf* population and shows a significant admixture between the Asian *Aaa* and the African *Aaf* in the coast.

Figure 4: Principal component analysis (PCA) on: A) the Kenyan *Ae. aegypti* populations and B) the broad global dataset. PCA was implemented and plotted in R. ggplot package, presenting the projection of the first two PCs. Populations originating from different regions are presented with different colors as shown in the inset.

Pearson correlation test for the *Aaa* ancestry with the Kenyan population shows a correlation coefficient (R^2) of 0.69 which indicates a strong negative correlation (Fig. 5). There is a negative correlation between the ancestry of *Ae. aegypti* and the distance from Mombasa. There is a negative correlation (Fig 5; R^2= 0.69 and p-value of 2.89e-18) between the ancestry of Ae. aegypti and the distance from Mombasa among the Kenyan populations. As the distance from Mombasa increases, the ancestry proportion of *Aaa* decreases.

Figure 5: Correlation test between the *Aaa* ancestry and the Kenyan populations. X-axis: Distance from Mombasa (in kilometers), Y-axis: Ancestry proportion of *Ae. aegypti aegypti*. Gray circles represent the ancestry data of *Ae. aegypti* from different locations in Kenya. The red line is the best-fit regression line showing the trend. R^2 value: 0.69, indicating that 69% of the variance in ancestry. P-value: 2.89e-18, indicating a highly significant correlation.

Genetic diversity and differentiation

The pairwise estimates of genetic differentiation among the six geographical populations ranged from 0.014 between Malaba and Eldoret to 0.207 between Mombasa and Malaba (Table 1). Consistent with Admixture results, Mombasa and Mariakani form a closely related group with F_{ST} < 0.05. The western region showed no differentiation within, the closest population pair with Mariakani population was Kisumu (F_{ST} = 0.088). Pairwise F_{ST} values among these study populations indicated that coastal populations had lower mean F_{ST} values compared to western populations. The population pairs Mombasa-Malaba had the highest F_{ST} values (0.207) observed followed by Mombasa – Eldoret. There was a lower degree of genetic differentiation among coastal and western populations of Kenya. Mean genetic diversity estimates over loci for each mosquito population are summarized in Table 2.

Analysis of molecular variation (AMOVA) indicated significant molecular variation within the individual populations compared to among populations. Genetic variation within individual population was 82%, 11% among individuals and 7% among populations (Table 3). AMOVA on the dataset indicates that most of the variation can be explained at the individual level, with a lower contribution from the population level.

Table 1: Pairwise F_{ST} values between the Kenyan populations. All pairwise F_{ST} estimations were significant at significance level p<0.005 (Supplimentary3 (S3).

	Mariakani	Malaba	Nakuru	Eldoret	Mombasa	Kisumu
Mariakani	0	0.138	0.084	0.117	0.049	0.088
Malaba	0.138	0	0.06	0.014	0.207	0.028
Nakuru	0.084	0.06	0	0.054	0.132	0.038
Eldoret	0.117	0.014	0.054	0	0.183	0.019
Mombasa	0.049	0.207	0.132	0.183	0	0.131
Kisumu	0.088	0.028	0.038	0.019	0.131	0

Table 2: Summary of the mean genetic diversity indices over loci for each of *Aedes aegypti* mosquito populations

**Region**	**Population**	**Num**	**Eff-Num**	**Ho**	**Hs**	**Gis**
**Coastal Kenya**	Mombasa	1.807	1.448	0.243	0.28	0.131
	Mariakani	1.806	1.409	0.225	0.262	0.143
**Western Kenya**	Nakuru	1.749	1.385	0.224	0.245	0.086

	Eldoret	1.715	1.358	0.205	0.231	0.114
	Kisumu	1.797	1.393	0.217	0.252	0.136
	Malaba	1.727	1.351	0.197	0.224	0.121

Num, Number of alleles, Eff-Num, Effective number of alleles, Ho, Observed Heterozygosity, Hs, Heterozygosity Within Populations, Gis, Inbreeding coefficient

Table 3: AMOVA of genetic variation in *Ae. aegypti* samples collected from different sites in Kenya

Source of variation	%Var	F-stat	F-value	Std.Dev	c.i.2.5%	c.i.97.5%	P-value	F'-value
**Within individuals**	0.816	F_it	0.184	0.002	0.18	0.187		
**Among individuals**	0.111	F_is	0.12	0.002	0.116	0.123	0.001	
**Among populations**	0.073	F_st	0.073	0.001	0.071	0.074	0.001	0.097

Isolation by distance

Genetic distance (F_{ST}) of all six *Ae. aegypti* populations in Kenya was positively correlated with geographic distance (Observation = 0.869, p = 0.0023), indicating isolation by distance (IBD; Fig. 6). The genetic differentiation between *Ae. aegypti* populations showed a clear signature of IBD.

Figure 6: Isolation-by-distance plots for all pairs of populations from Kenya. Statistical significance was evaluated through a Mantel test as implemented in the "ade4" R package yielding a significant positive slope. The original value of the correlation between the two matrices (geographic distance and genetic distance) represented by dot. Genetic distance is given as the linearized F_{ST} and geographic distance is provided in kilometers (Km).

Phylogenetic analysis

We evaluated the relationships among populations of *Ae. aegypti*, as well as other populations of the species group using maximum-likelihood inference on the population-level SNP data set. The phylogeny constructed from the SNP data shows that mosquitoes from western Kenya formed a clade within the Africa population (Fig.7). All populations outside Africa form a monophyletic group distinct from all the African populations. Consistent with their admixture patterns, coastal populations are placed in an intermediate position between the *Aaa* lineage and the other Kenyan and African populations. The Nakuru, Eldoret, Kisumu and Malaba populations cluster together with the *Aaf* from Africa.

Figure 7: Maximum likelihood (ML) annotated phylogenetic tree re-constructed using a panel of ~22,849 SNPs. *Aaa* samples from New World and Asia were used to test the distinctiveness of *Aaf* and *Aaa* lineages (for details on branch/node labels see S1 Table2).

Discussion

The *Ae. aegypti* mosquito is widely distributed throughout tropical and subtropical regions of the world. Global trade and international travel, as well as climate change, have considerably accelerated the spread of these vectors to new countries thus elevating the risk of disease transmission. This is evident in the analysis of Kenyan *Ae. aegypti* populations that shows evidence of gene flow of the invasive *Aaa* subspecies into Kenyan native *Aaf* . This invasion raises the possibility of disease epidemics in areas where they are not endemic, thus emphasizing the importance of population structure and genetic studies to identify regional genetic variants in mosquito populations. Studies have shown that the globally invasive *Aedes aegypti* populations have enhanced transmission of the majority of emerging mosquito-borne viruses such as Zika and dengue (58).

Population genetics analyses of the Kenyan *Ae. aegypti* using SNPs genetic markers (~, 22,849 SNPs) revealed two genetically distinct groups within the study populations, one in coastal and the others in western regions (Fig 2i). The western region is genetically homogenous and clusters with native African subspecies, with minimal presence of admixture between the two subspecies. The observed admixture signature in the coast is likely the result of human-mediated passive introductions to the coastal region via the shipping cargo ships from other continents. Passive human-mediated dispersal is the main cause of invasion of vectors as observed in other studies (59, 60). The Kenyan coastal populations are genetically close to *Aaa* outside Africa, suggesting gene flow and invasion driven by long dispersal event that led to establishment of foreign subspecies locally. Our results agree with previous studies showing possible hybrids in the coastal populations of Kenya most likely resulting from the introduction of *Aaa* from the Asian continent (22, 61).

The presence of the two subspecies in urban rural areas supports prior studies based on microsatellites that evaluated the spatial distribution of *Ae. aegypti* subspecies in Kenya (62, 63). In this study, we collected *Aaf* from urban areas in Western Kenya (Nakuru, Eldoret, Kisumu and Malaba) and a few in Kilifi in coastal region. *Aedes aegypti* populations in Africa are known to historically breed almost entirely in forests. Today, the vector in Africa can be found in urban habitats as observed in previous studies (22, 64, 65). We observed the foreign genetic signature at high frequency in the Kenyan coastal region, with frequency decreasing as distance from the sea to the mainland, in agreement with the previous studies (63). Western Kenya had lower presence of *Aaa* ancestry. The presence of native population in urban/cities is the result of increased urbanization, with human settlements encroaching into forests. This observation concurs with previous studies that observed high abundance of the vector in urban areas (7, 9). Such an increase

in urbanization in African urban areas could confer a greater adaptive advantage for *Aaa* over *Aaf* since it is adapted to urban environments.

The observed clustering of coastal populations with Asian population samples is likely the result of historical trade between the two continents. The slave trade likely led to the introduction of the foreign *Aedes* populations to the East African coast in the early 1900s which is now interbreeding with the native population (20, 29). Admixture may complicate efforts to control *Ae. aegypti* by shifting typical behaviors associated with this mosquito in a way that may allow them to evade control measures that used to be effective against them (66). The Kenyan coastal region had sporadic outbreaks of dengue and chikungunya virus in the past but is now becoming endemic for both emerging diseases; this may be attributed to the high human bite-rate of the hybrid species of *Ae. aegypti* observed in the coastal region (67), and its demonstrated high competence compared to native *Aaf* (Aubry 2020). Prior studies of Kenyan outbreaks indicated that the viruses could have originated in South East Asia (68, 69). This was also evident in our study which showed that the Asian populations are a likely source of *Aaa* invasion into Kenya, possibility though importation of infected mosquitoes/eggs. The availability of highly competent vectors has amplified arbovirus outbreaks in the Kenyan coastal region. The detection of dengue and chikungunya in sero-prevalence studies in mainland cities and towns is an indicator that the infections are spreading to the mainland as evident in parts of western Kenya (70, 71). This low transmission is likely due to the presence of less efficient subspecies of *Aaf* vectors observed in those regions (58). .

Genetic patterns observed in the study suggest that the coastal *Ae. aegypti* populations in study cities are admixed. This is in contrast with previous studies where the presence of the two

subspecies coexisting sympatrically in peri-domestic and sylvan populations in coastal Kenya was observed (29). The distribution of the two subspecies of *Ae. agypti* in Rabai in coastal region was observed where the species were closely breeding in the same ecosystem. Historical literature from the 1960s suggests that the two subspecies remained distinct in Rabai (4) for some time but formed mixed hybrid swarms along the coast. This is highly consistent with our findings. It is not clear why the two coexisted for so long in the Rabai environment without mixing, but mixed more freely just tens of kilometers away. Up-to-date the 'pure' *Aaa* have not been found along the coast since around 2010 (72) . The present study highlights the complex patterns of vector colonization that have occurred in Kenya. Previous studies speculated that the presence of *Aaf* in urban areas rather than in rural/forest areas which are typical for the species, was due to the increased urbanization and human activities such as deforestation (73). The observed admixed population in the coastal region, most likely reflects the historical relationship of the Kenyan coastal region with the Asian and European region during the slave trade and continuous trade between the two continents. The Arab and Asian traders used the coastal region as a port-of-call for ship provisioning during travels between Europe and the African continent and also as a slave outpost for the Atlantic slave trade (74). The considerable movement of ships/trade between the islands and both continents could have provided ample opportunity for the introduction of the foreign mosquito species. Mombasa is a tourist destination area allowing influx of tourists from all over the world, some from endemic areas for known viruses; these may potentially introduce viruses that are known to be transmitted by the *Ae. aegypti*. The introduction of these pathogens may place the native communities in danger due to observed high hybridization between the two subspecies that may promote a more opportunistic feeding behavior thus increasing the chance for the accidental transmission of viruses to humans.

Conclusion

Human economic activity is the primary cause of *Ae. aegypti* invasion into foreign countries as observed by the presence of *Aaa* ancestry in mosquito populations from coastal Kenya. Human-mediated transportation and migration are facilitating long distance vector dispersal and could drive admixture of forest-adapted and urban-adapted populations leading to increased adaptive flexibility, with implications for disease transmission and control. Effective, efficient, and sustainable control of invasive mosquitoes, such as *Ae. Aegypti* and identification of the routes of introduction and dispersal will be key to prevent future incursions and assess their potential health threat.

Acknowledgements

We recognize the technical support of Viral Hemorrhagic Fever Lab team members (KEMRI, Nairobi). We are grateful to Samuel Owaka, GIS support unit, KEMRI for producing the map of the study area. We are also grateful for the support from the county staff, local chiefs as well as community members of Mombasa, Kilifi, Nakuru, Eldoret, Kisumu and Busia Counties. Sample processing and genotyping was supported by NIH NIAID R01 AI101112 awarded to J.R. Powell.

Authors' contributions

FM carried out part of the molecular laboratory work, the data analyses, and drafted the manuscript. DB supported the data analyses, edited and reviewed the manuscript. AG-S carried out part of the molecular work, supervised the data analyses, and revised the manuscript critically for important intellectual content. All authors revised the manuscript critically for important

intellectual content. JL conceived, designed, and coordinated the study, reviewed the manuscript, and obtained funds for the research.

Competing Interests

The authors have declared that no competing interests exist

SUPPLEMENTARY MATERIAL

Supplementary Table SI: Kenyan *Aedes aegypti* populations.

Supplementary Table S2: Aedes aegypti world and Kenyan populations included in the phylogenetic analysis

Supplementary S2: Admixture analysis of *Ae. aegypti* at the global data set

Supplementary S3: Pairwise FST significant values for the Kenyan populations

References

1. Kraemer MU, Sinka ME, Duda KA, Mylne AQ, Shearer FM, Barker CM, et al. The global distribution of the arbovirus vectors Aedes aegypti and Ae. albopictus. elife. 2015;4:e08347.
2. Mattingly P. Genetical aspects of the Aedes aegypti problem: I.—taxonomy and bionomics. Annals of Tropical Medicine & Parasitology. 1957;51(4):392-408.
3. Powell JR, Gloria-Soria A, Kotsakiozi P. Recent history of Aedes aegypti: Vector genomics and epidemiology records. Bioscience. 2018;68(11):854-60.
4. Tabachnick WJ, Powell JR. Genetic structure of the East African domestic populations of Aedes aegypti. Nature. 1978;272(5653):535-7.
5. Tchouassi DP, Agha SB, Villinger J, Sang R, Torto B. The distinctive bionomics of Aedes aegypti populations in Africa. Curr Opin Insect Sci. 2022;54:100986.
6. Kraemer MU, Sinka ME, Duda KA, Mylne AQ, Shearer FM, Barker CM. The global distribution of the arbovirus vectors Aedes aegypti and Ae. albopictus. 2015;4:e08347.
7. Gubler DJ. Dengue, urbanization and globalization: the unholy trinity of the 21st century. Tropical medicine and health. 2011;39(4SUPPLEMENT):S3-S11.
8. McMichael AJ. Environmental and social influences on emerging infectious diseases: past, present and future. Philos Trans R Soc Lond B Biol Sci. 2004;359(1447):1049-58.
9. Xia S, Dweck HK, Lutomiah J, Sang R, McBride CS, Rose NH, et al. Larval sites of the mosquito Aedes aegypti formosus in forest and domestic habitats in Africa and the potential association with oviposition evolution. Ecology and evolution. 2021;11(22):16327-43.
10. Rose NH, Badolo A. Dating the origin and spread of specialization on human hosts in Aedes aegypti mosquitoes. 2023;12.

11. Konongoi L, Ofula V, Nyunja A, Owaka S, Koka H, Makio A, et al. Detection of dengue virus serotypes 1, 2 and 3 in selected regions of Kenya: 2011–2014. Virology journal. 2016;13(1):1-11.
12. Blaylock JM, Maranich A, Bauer K, Nyakoe N, Waitumbi J, Martinez LJ, et al. The seroprevalence and seroincidence of dengue virus infection in western Kenya. Travel medicine and infectious disease. 2011;9(5):246-8.
13. Leaf-nosed bat. Encyclopædia Britannica: Encyclopædia Britannica Online; 2009.
14. Sang R, Lutomiah J, Chepkorir E, Tchouassi DP. Evolving dynamics of Aedes-borne diseases in Africa: a cause for concern. Curr Opin Insect Sci. 2022;53:100958.
15. Lutomiah J, Barrera R, Makio A, Mutisya J, Koka H, Owaka S, et al. Dengue outbreak in Mombasa city, Kenya, 2013–2014: entomologic investigations. PLoS neglected tropical diseases. 2016;10(10):e0004981.
16. Agha SB, Tchouassi DP, Bastos AD, Sang R. Assessment of risk of dengue and yellow fever virus transmission in three major Kenyan cities based on Stegomyia indices. PLoS neglected tropical diseases. 2017;11(8):e0005858.
17. Agha SB, Tchouassi DP, Bastos AD, Sang R. Dengue and yellow fever virus vectors: seasonal abundance, diversity and resting preferences in three Kenyan cities. Parasites & vectors. 2017;10(1):1-10.
18. Gloria-Soria A, Ayala D, Bheecarry A, Calderon-Arguedas O, Chadee DD, Chiappero M, et al. Global genetic diversity of Aedes aegypti. Molecular ecology. 2016;25(21):5377-95.
19. Rose NH, Sylla M, Badolo A, Lutomiah J, Ayala D, Aribodor OB, et al. Climate and Urbanization Drive Mosquito Preference for Humans. Current biology : CB. 2020;30(18):3570-9.e6.
20. Brown JE, Evans BR, Zheng W, Obas V, Barrera-Martinez L, Egizi A, et al. Human impacts have shaped historical and recent evolution in Aedes aegypti, the dengue and yellow fever mosquito. Evolution. 2014;68(2):514-25.
21. McBride CS, Baier F, Omondi AB, Spitzer SA, Lutomiah J, Sang R, et al. Evolution of mosquito preference for humans linked to an odorant receptor. Nature. 2014;515(7526):222-7.
22. Kotsakiozi P, Evans BR, Gloria-Soria A, Kamgang B, Mayanja M, Lutwama J, et al. Population structure of a vector of human diseases: Aedes aegypti in its ancestral range, Africa. Ecology and evolution. 2018;8(16):7835-48.
23. Powell JR, Tabachnick WJ. History of domestication and spread of Aedes aegypti-a review. Memórias do Instituto Oswaldo Cruz. 2013;108:11-7.
24. Xia S, Cosme LV, Lutomiah J, Sang R, Ngangue MF, Rahola N, et al. Genetic structure of the mosquito Aedes aegypti in local forest and domestic habitats in Gabon and Kenya. Parasites & Vectors. 2020;13(1):1-13.
25. Redmond SN, Sharma A, Sharakhov I, Tu Z, Sharakhova M, Neafsey DE. Linked-read sequencing identifies abundant microinversions and introgression in the arboviral vector Aedes aegypti. BMC Biol. 2020;18(1):26.
26. Hartberg W, Craig Jr G. Reproductive isolation in Stegomyia mosquitoes. II. Hybrid breakdown between Aedes aegypti and A. mascarensis. Evolution. 1970:692-703.
27. Soghigian J, Gloria-Soria A, Robert V, Le Goff G, Failloux AB, Powell JR. Genetic evidence for the origin of Aedes aegypti, the yellow fever mosquito, in the southwestern Indian Ocean. Molecular Ecology. 2020;29(19):3593-606.

28. Lambrechts L. Quantitative genetics of Aedes aegypti vector competence for dengue viruses: towards a new paradigm? Trends in parasitology. 2011;27(3):111-4.
29. Brown JE, McBride CS, Johnson P, Ritchie S, Paupy C, Bossin H, et al. Worldwide patterns of genetic differentiation imply multiple 'domestications' of Aedes aegypti, a major vector of human diseases. Proceedings of the Royal Society B: Biological Sciences. 2011;278(1717):2446-54.
30. Gloria-Soria A, Brown JE, Kramer V, Hardstone Yoshimizu M, Powell JR. Origin of the dengue fever mosquito, Aedes aegypti, in California. PLoS Neglected Tropical Diseases. 2014;8(7):e3029.
31. Evans BR, Gloria-Soria A, Hou L, McBride C, Bonizzoni M, Zhao H, et al. A Multipurpose, High-Throughput Single-Nucleotide Polymorphism Chip for the Dengue and Yellow Fever Mosquito, Aedes aegypti. G3 (Bethesda, Md). 2015;5(5):711-8.
32. Edwards FW. Mosquitoes of the Ethiopian Region. HI.-Culicine Adults and Pupae. Mosquitoes of the Ethiopian Region HI-Culicine Adults and Pupae. 1941.
33. Jupp PG. Mosquitoes of Southern Africa: Culicinae and Toxorhynchitinae: Ekogilde Publishers; 1996.
34. Rueda LM. Pictorial keys for the identification of mosquitoes (Diptera: Culicidae) associated with dengue virus transmission. Walter Reed Army Inst Of Research Washington Dc Department Of Entomology, 2004.
35. Evans BR, Gloria-Soria A, Hou L, McBride C, Bonizzoni M, Zhao H, et al. A multipurpose, high-throughput single-nucleotide polymorphism chip for the dengue and yellow fever mosquito, Aedes aegypti. G3: Genes, Genomes, Genetics. 2015;5(5):711-8.
36. Kotsakiozi P, Gloria-Soria A, Schaffner F, Robert V, Powell JR. Aedes aegypti in the Black Sea: recent introduction or ancient remnant? Parasites & vectors. 2018;11:1-13.
37. Gómez-Palacio A, Morinaga G, Turner PE, Micieli MV, Elnour M-AB, Salim B, et al. Robustness in population-structure and demographic-inference results derived from the Aedes aegypti genotyping chip and whole-genome sequencing data. G3: Genes, Genomes, Genetics. 2024;14(6):jkae082.
38. Gloria-Soria A, Lima A, Lovin DD, Cunningham JM, Severson DW, Powell JR. Origin of a high-latitude population of Aedes aegypti in Washington, DC. The American journal of tropical medicine and hygiene. 2018;98(2):445.
39. Purcell S, Chang C. PLINK. www. cog-genomics. org/plink/1.9. Accessed; 2021.
40. Chang CC, Chow CC, Tellier LC, Vattikuti S, Purcell SM, Lee JJ. Second-generation PLINK: rising to the challenge of larger and richer datasets. Gigascience. 2015;4(1):s13742-015-0047-8.
41. Purcell S, Chang C. Plink 1.9 package. 2018. URL https://www cog-genomics org/plink/19. 2019;563.
42. Raj A, Stephens M, Pritchard JK. fastSTRUCTURE: variational inference of population structure in large SNP data sets. Genetics. 2014;197(2):573-89.
43. Kopelman NM, Mayzel J, Jakobsson M, Rosenberg NA, Mayrose I. Clumpak: a program for identifying clustering modes and packaging population structure inferences across K. Molecular ecology resources. 2015;15(5):1179-91.
44. Rosenberg NA. DISTRUCT: a program for the graphical display of population structure. Molecular ecology notes. 2004;4(1):137-8.

45. Earl DA, VonHoldt BM. STRUCTURE HARVESTER: a website and program for visualizing STRUCTURE output and implementing the Evanno method. Conservation genetics resources. 2012;4:359-61.
46. Team RC. R: A language and environment for statistical computing. R Foundation for Statistical Computing, Vienna, Austria. Available online at https://www. R-project. org.
47. Team RC. R Core Team R: a language and environment for statistical computing. Foundation for Statistical Computing. 2020.
48. Meirmans P, Amsterdam IV. GenoDive. 2019.
49. Meirmans PG. genodive version 3.0: Easy-to-use software for the analysis of genetic data of diploids and polyploids. Molecular Ecology Resources. 2020;20(4):1126-31.
50. Lischer HE, Excoffier L. PGDSpider: an automated data conversion tool for connecting population genetics and genomics programs. Bioinformatics. 2012;28(2):298-9.
51. Lischer H, Excoffier L, Spider P. An automated data conversion tool for connecting population genetics and genomics programs. Bioinformatics. 2012;28:298-9.
52. Excoffier L, Smouse PE, Quattro J. Analysis of molecular variance inferred from metric distances among DNA haplotypes: application to human mitochondrial DNA restriction data. Genetics. 1992;131(2):479-91.
53. Dray S, Dufour A-B. The ade4 package: implementing the duality diagram for ecologists. Journal of statistical software. 2007;22:1-20.
54. Ersts P. Internet 2016 Geographic Distance Matrix Generator (version 1.2. 3). American Museum of Natural History. Center for Biodiversity and Conservation Available from http://biodiversityinformatics amnh org/open_source/gdmg Accessed on Feb. 2016;2:2016.
55. Nguyen L-T, Schmidt HA, Von Haeseler A, Minh BQ. IQ-TREE: a fast and effective stochastic algorithm for estimating maximum-likelihood phylogenies. Molecular biology and evolution. 2015;32(1):268-74.
56. Hoang DT, Chernomor O, Von Haeseler A, Minh BQ, Vinh LS. UFBoot2: improving the ultrafast bootstrap approximation. Molecular biology and evolution. 2018;35(2):518-22.
57. Evanno G, Regnaut S, Goudet J. Detecting the number of clusters of individuals using the software STRUCTURE: a simulation study. Mol Ecol. 2005;14(8):2611-20.
58. Dabo S, Henrion-Lacritick A, Lecuyer A, Jiolle D, Paupy C, Ayala D, et al. Extensive variation and strain-specificity in dengue virus susceptibility among African Aedes aegypti populations. 2024;18(3):e0011862.
59. Pless E, Gloria-Soria A, Evans BR, Kramer V, Bolling BG, Tabachnick WJ, et al. Multiple introductions of the dengue vector, Aedes aegypti, into California. PLoS neglected tropical diseases. 2017;11(8):e0005718.
60. Kotsakiozi P, Gloria-Soria A, Caccone A, Evans B, Schama R, Martins AJ, et al. Tracking the return of Aedes aegypti to Brazil, the major vector of the dengue, chikungunya and Zika viruses. PLoS neglected tropical diseases. 2017;11(7):e0005653.
61. Redmond SN, Sharma A, Sharakhov I, Tu Z, Sharakhova M, Neafsey DE. Linked-read sequencing identifies abundant microinversions and introgression in the arboviral vector Aedes aegypti. BMC biology. 2020;18(1):1-14.
62. Futami K, Iwashita H, Higa Y, Lutiali P, Sonye G, Mwatele C, et al. Geographical Distribution of Aedes aegypti aegypti and Aedes aegypti formosus (Diptera: Culicidae) in Kenya and Environmental Factors Related to Their Relative Abundance. Journal of Medical Entomology. 2020;57(3):772-9.

63. Powell JR, Tabachnick WJ. History of domestication and spread of Aedes aegypti--a review. Mem Inst Oswaldo Cruz. 2013;108 Suppl 1(Suppl 1):11-7.
64. Paupy C, Brengues C, Kamgang B, Hervé J-P, Fontenille D, Simard F. Gene flow between domestic and sylvan populations of Aedes aegypti (Diptera: Culicidae) in North Cameroon. Journal of medical entomology. 2014;45(3):391-400.
65. Rose NH, Sylla M, Badolo A, Lutomiah J, Ayala D, Aribodor OB, et al. Climate and urbanization drive mosquito preference for humans. bioRxiv. 2020.
66. Barton NH. The role of hybridization in evolution. Molecular ecology. 2001;10(3):551-68.
67. Ndenga BA, Mutuku FM, Ngugi HN, Mbakaya JO, Aswani P, Musunzaji PS, et al. Characteristics of Aedes aegypti adult mosquitoes in rural and urban areas of western and coastal Kenya. PloS one. 2017;12(12):e0189971.
68. Langat SK, Eyase FL, Berry IM, Nyunja A, Bulimo W, Owaka S, et al. Origin and evolution of dengue virus type 2 causing outbreaks in Kenya: Evidence of circulation of two cosmopolitan genotype lineages. Virus evolution. 2020;6(1):veaa026.
69. Eyase F, Langat S, Berry IM, Mulwa F, Nyunja A, Mutisya J, et al. Emergence of a novel chikungunya virus strain bearing the E1:V80A substitution, out of the Mombasa, Kenya 2017-2018 outbreak. PLoS One. 2020;15(11):e0241754.
70. Shah MM, Ndenga BA, Mutuku FM, Vu DM, Grossi-Soyster EN, Okuta V, et al. High Dengue Burden and Circulation of 4 Virus Serotypes among Children with Undifferentiated Fever, Kenya, 2014-2017. Emerg Infect Dis. 2020;26(11):2638-50.
71. Higa LDOS, Garcia MV, Rodrigues VDS, Junior PB, Barradas Pina FT, Barros JC, et al. Effects of cypermethrin, chlorpyrifos and piperonyl butoxide-based pour-on and spray acaricides on controlling the tick Rhipicephalus microplus. Systematic and Applied Acarology. 2019;24(2):278-86.
72. Gloria-Soria A, Ayala D, Bheecarry A, Calderon-Arguedas O, Chadee DD, Chiappero M, et al. Global genetic diversity of Aedes aegypti. 2016;25(21):5377-95.
73. Tchouassi DP, Agha SB, Villinger J, Sang R, Torto B. The distinctive bionomics of Aedes aegypti populations in Africa. Current Opinion in Insect Science. 2022:100986.
74. Austen RA. The 19th Century Islamic Slave Trade from East Africa (Swahili and Red Sea Coasts): A Tentative Census1. The Economics of the Indian Ocean Slave Trade in the Nineteenth Century: Routledge; 2013. p. 21-44.

Figure 1

Click here to access/download;Figure;Study site Map.tif

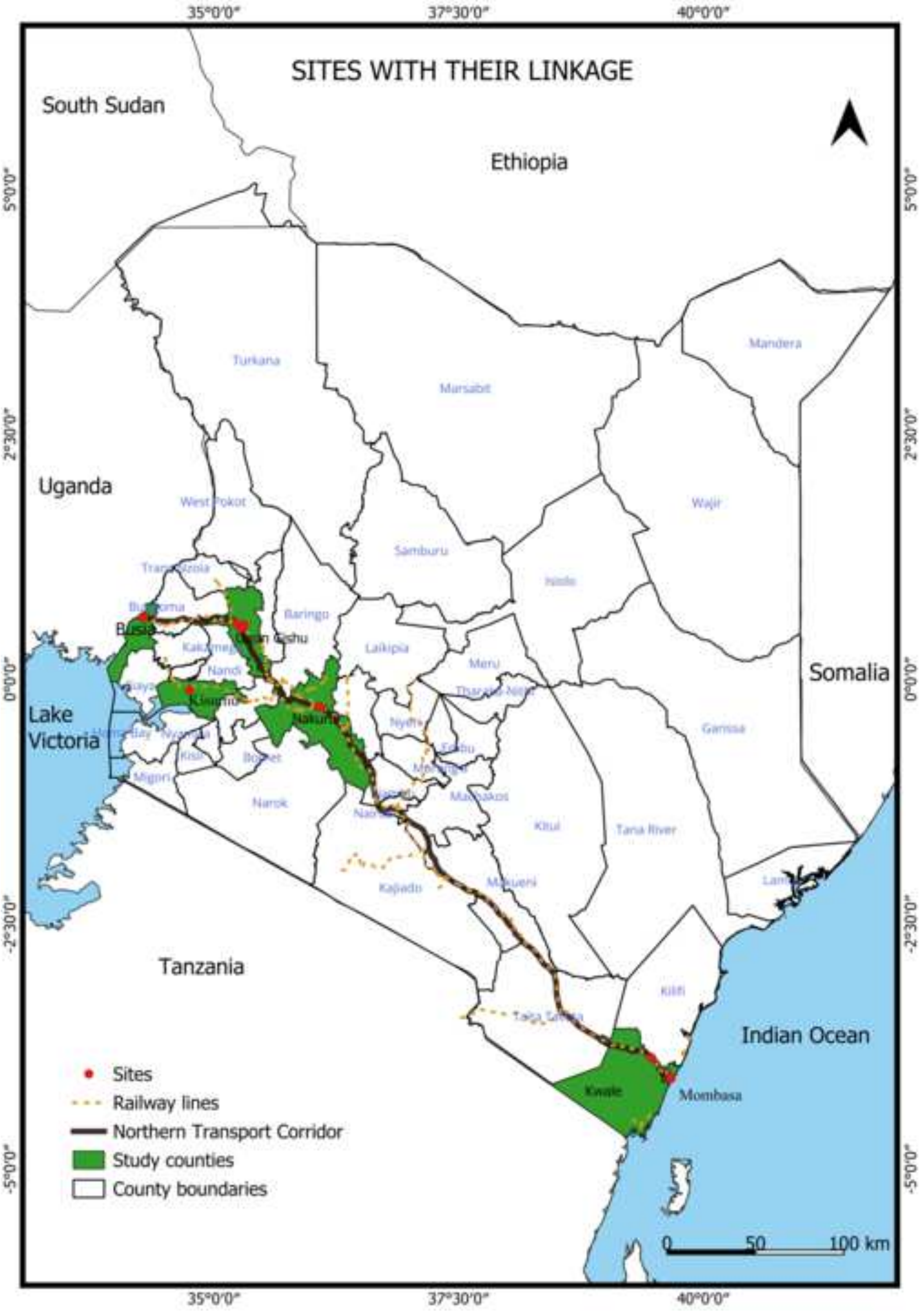

Figure 2

Click here to access/download;Figure;Population Structure for kenyan population.tif

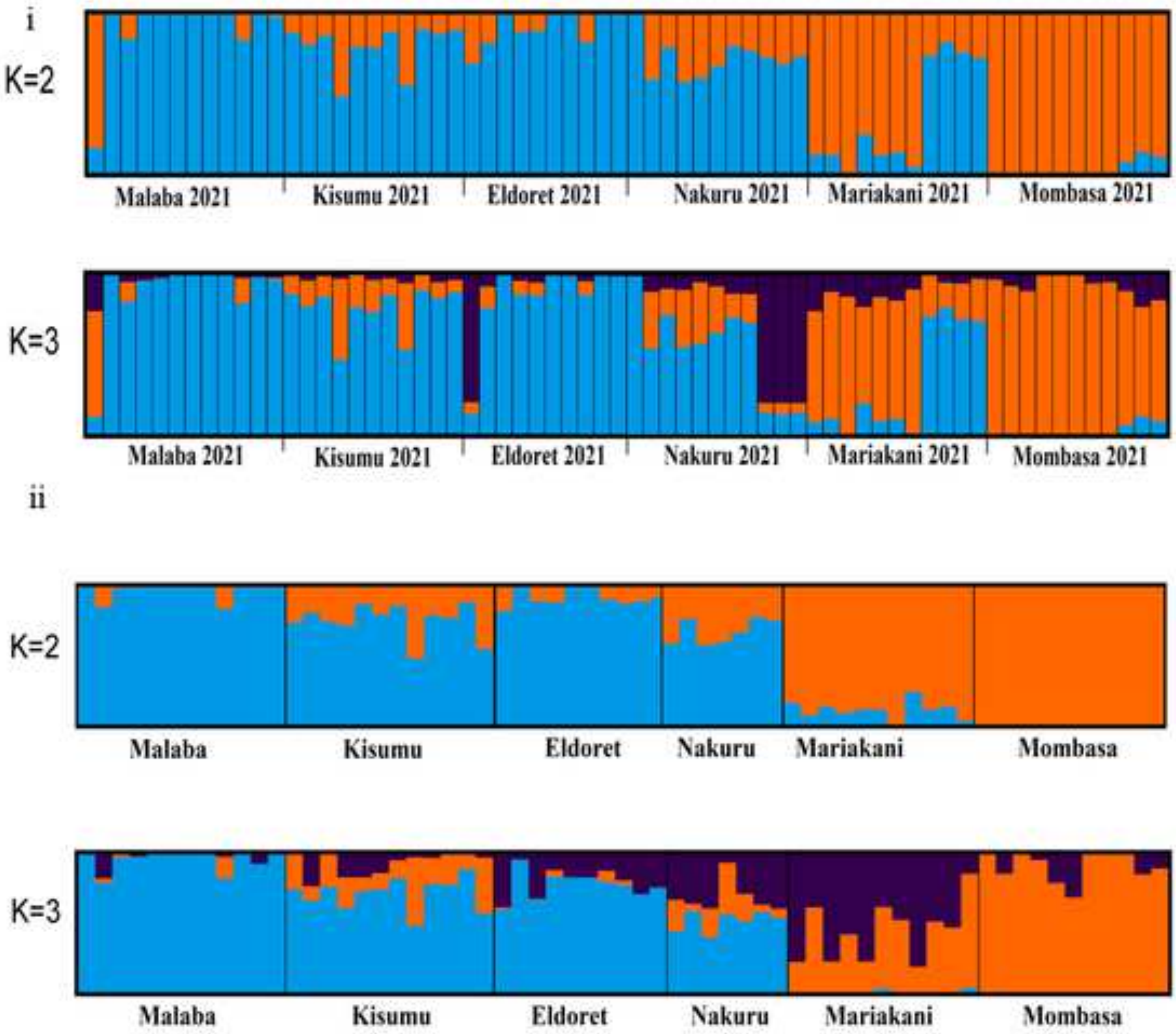

Figure 3

Click here to access/download;Figure;Global Admixture bar plots.tif

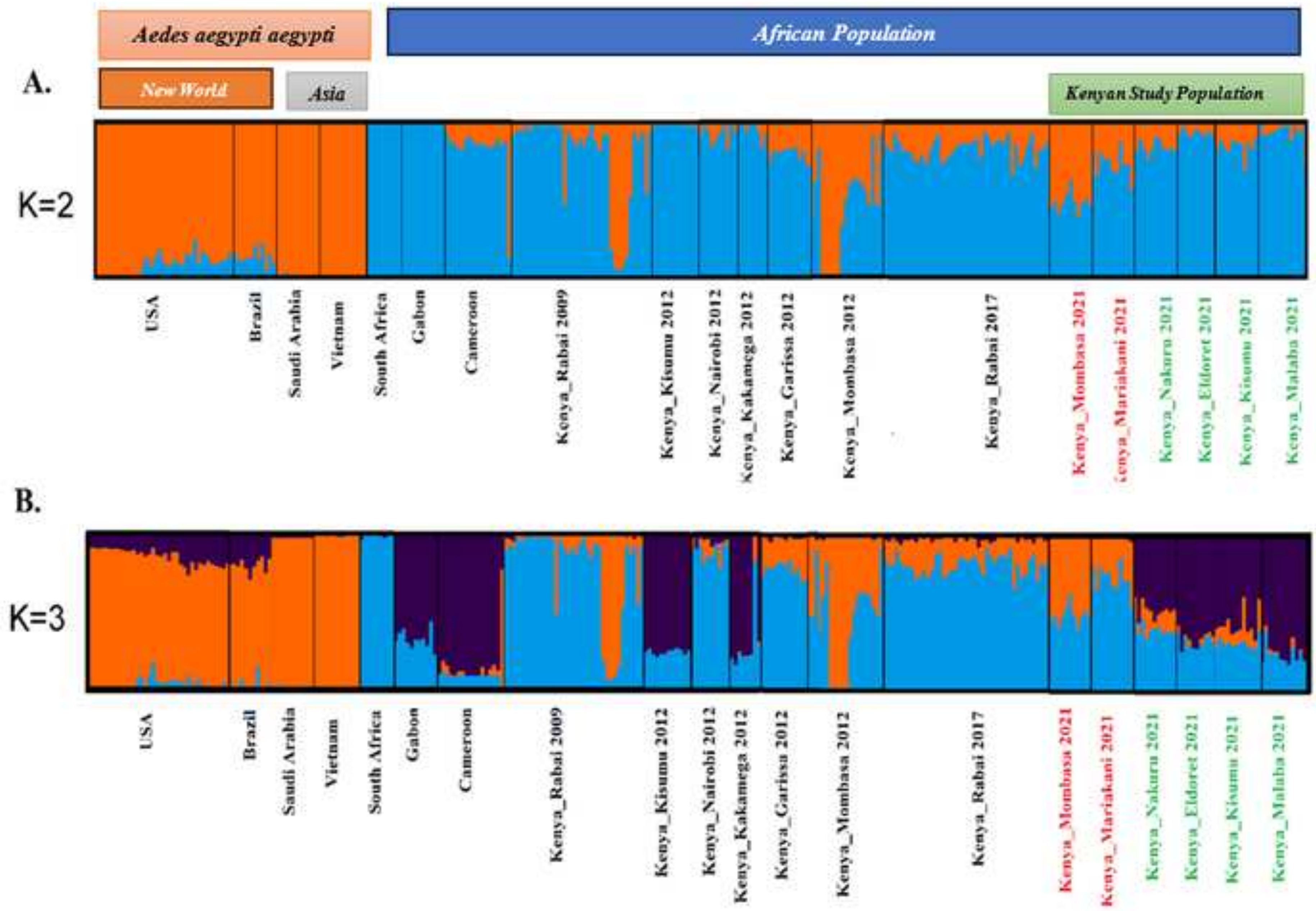

Figure 4

Click here to access/download;Figure;Principal component analysis (PCA).tif

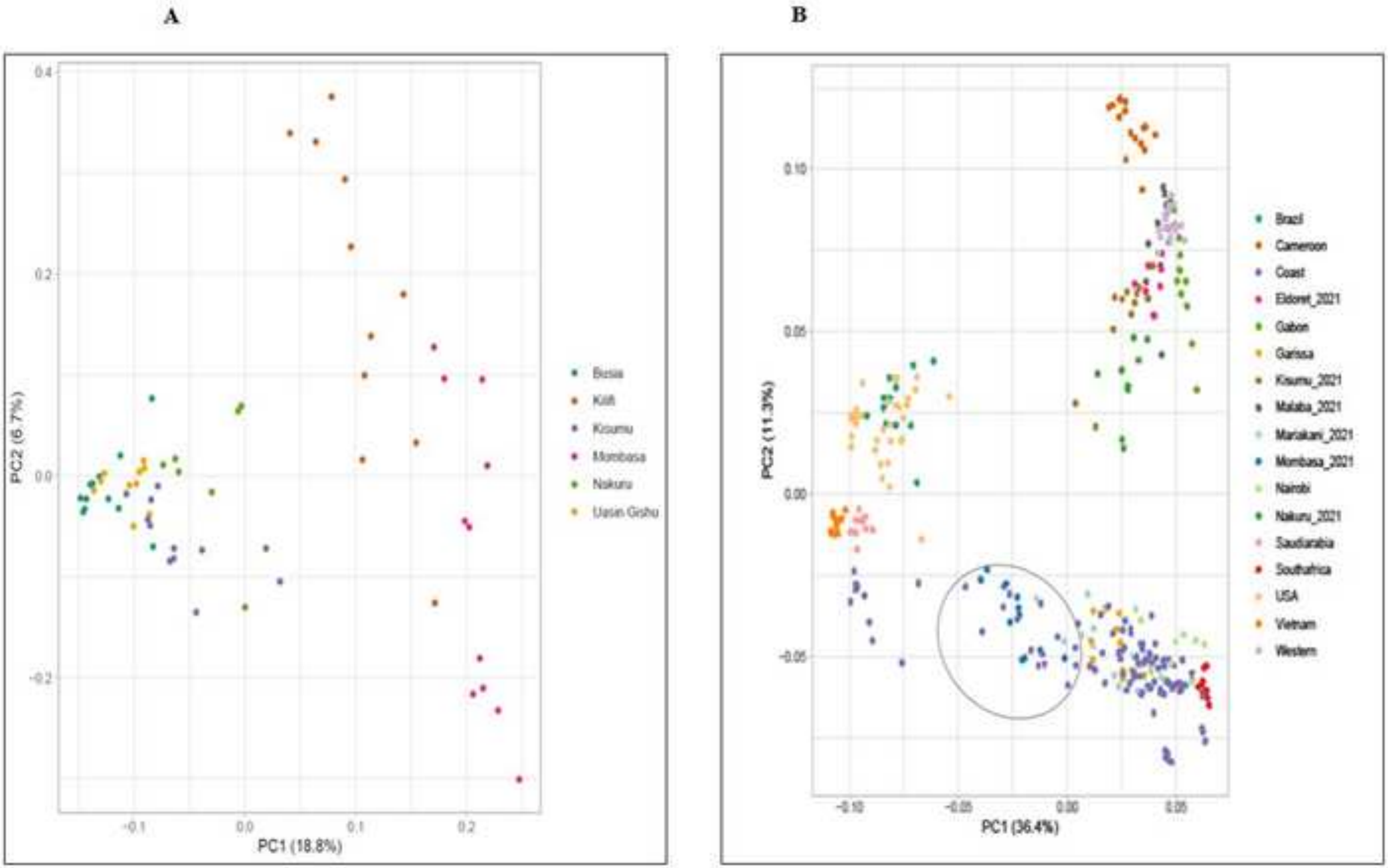

Click here to access/download;Figure;Correlation test.tif

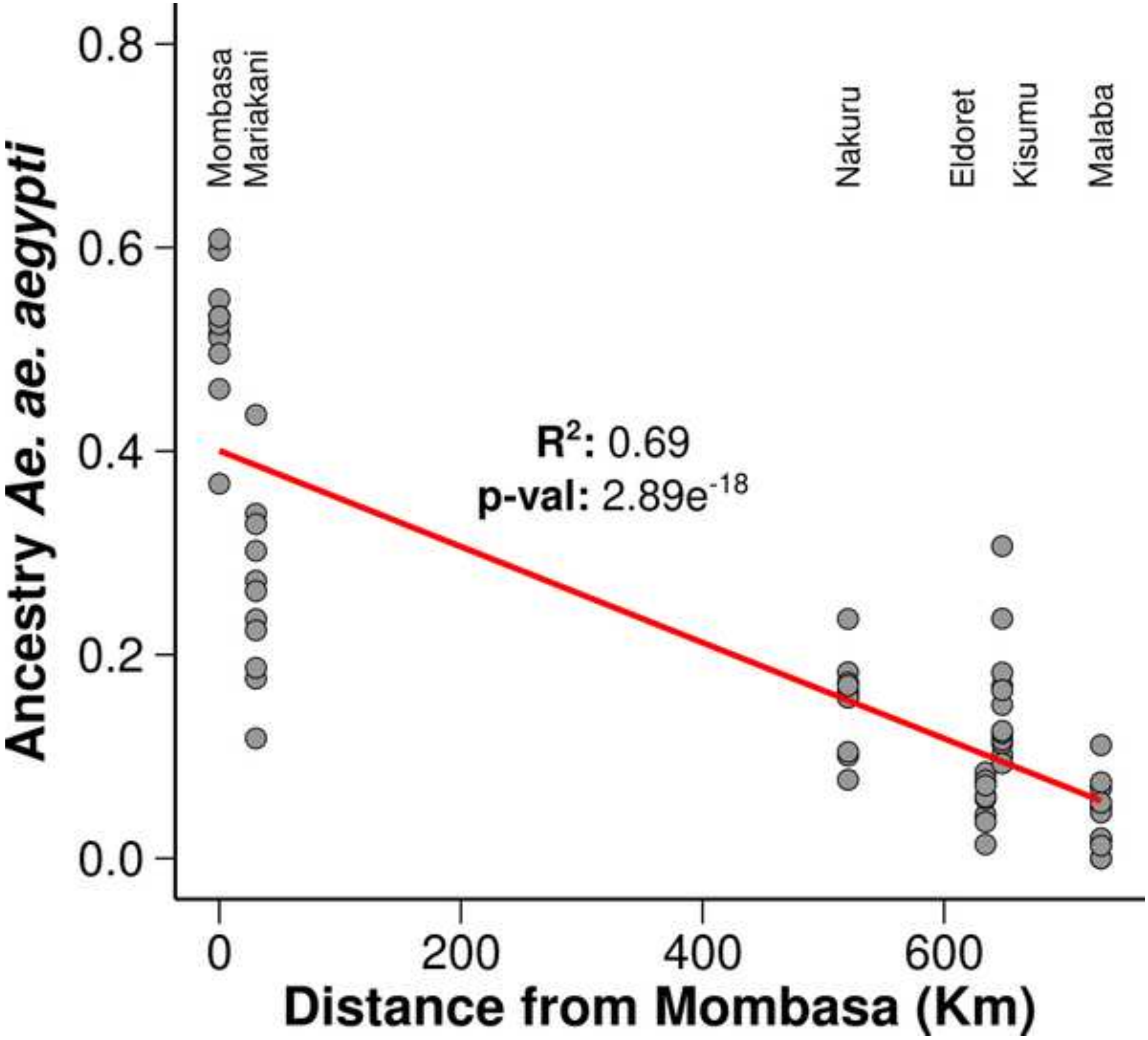

Figure 6

Click here to access/download;Figure;Isolation_by_Distance.tif

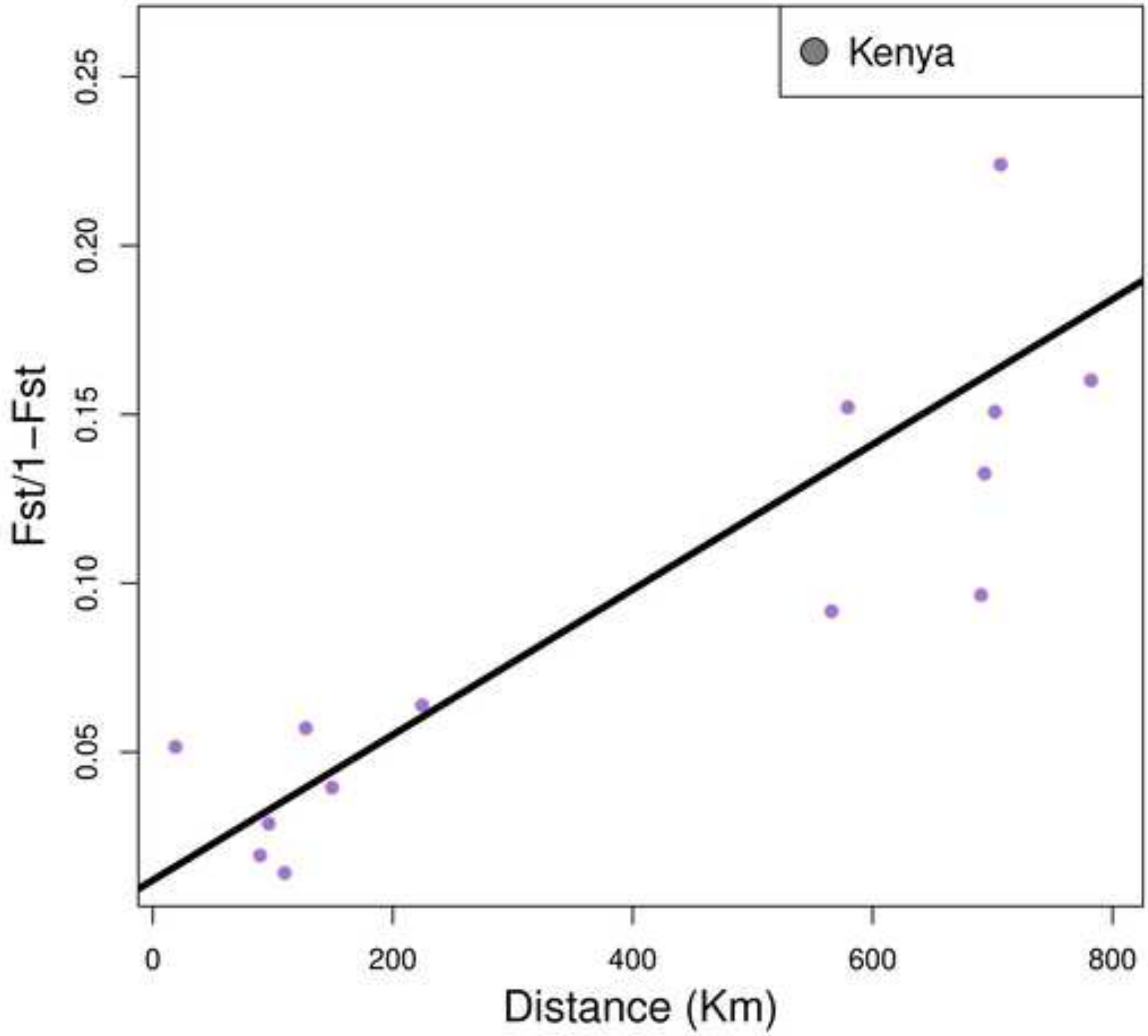

Figure 7

Click here to access/download;Figure;Maximum likelihood annotated phylogenetic tree.tif

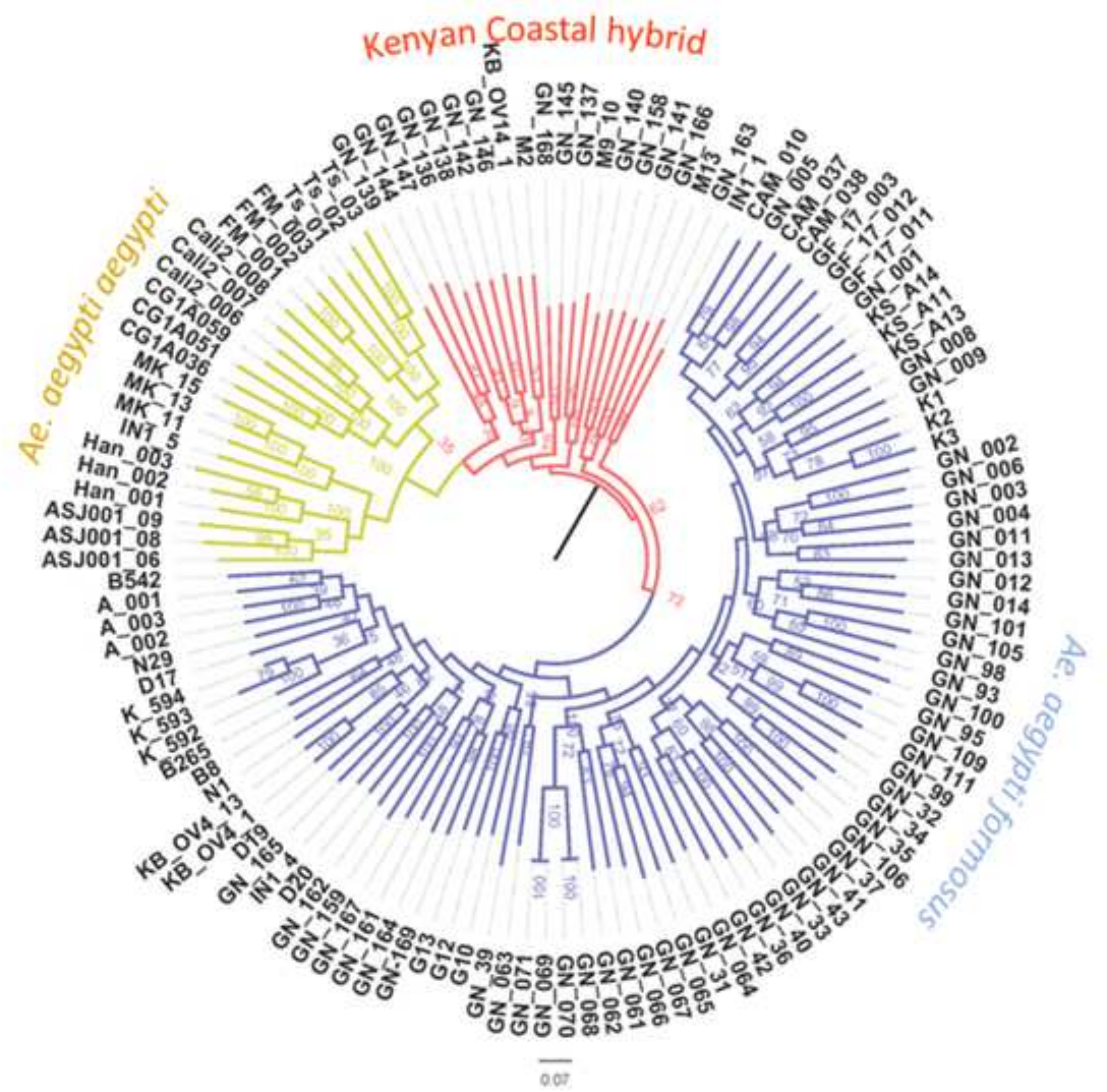

Click here to access/download
Supporting Information
Supplimentary 1.pdf

Click here to access/download
Supporting Information
Supplimentary 2.xlsx

Click here to access/download
Supporting Information
Supplimentary 3.pdf

Francis Mulwa Musili

KEMRI CVR

TO

THE EDITORS

PLOS NTD

RE: **Response to comments Raised by reviewers on the Manuscript**

Minor corrections

Line 150: How did the author screened SNPs using chip, in order to meet the Mendelian expectations.

Different concepts must be considered when designing an SNP chip, such as including only single-copy markers in the genome, ensuring they are inherited in a Mendelian fashion, and selecting markers associated through linkage disequilibrium due to evolutionary processes rather than physical proximity. All these concepts were considered by our group during the design of *Ae. aegypti* Axiom_aegypti1 platform and are detailed in Evans et al. (2015). As it is described in this paper, Mendelian inherence was studied by genotyping cohorts of F1 offspring and their respective parents, and the results showed that our custom platform contains probes for > 25,000 well-validated biallelic SNPs. In our current work we have eliminated those SNPs that Evans et al. (2015) found not to follow Mendelian inheritance patterns. We thank the reviewer for bringing this point to our attention. A section describing the SNP chip was missing from the original submission due to an oversight and has now been added starting lines 271, under a new section labeled "**Single Nucleotide Polymorphisms (SNPs) genotyping,** which provides more details on the genotyping platform. **Kindly refer to line 148-175 of the clean copy**

Line 152: the author should avoid vague expression such as …to eliminate SNPs .which did not genotype well, instead ,use technical and appropriate expressions such as … to eliminate missing data at those specific locations on the genome .Or simply state that … to eliminate missing data which did not confidently allow to identify the genetic variation at those SNP sites.

Well noted the sentence has been rephrased, kindly refer to line 171-173 of the clean copy

The statistical data analysis methods section is missing in this paper

We thank the reviewer for pointing out it. We have added more detailed information on the correlation test (lines 285-290.) and additional file for the pairwise test provided.

Major corrections

Line 109. Methods. The author should include a section on data quality control/check

We have added a citation describing the aegypti SNP chip development (Evans et al. 2015). Futhermore, the manuscript now includes additional information regarding the removal of samples that do not meet the quality requirements as part of the Single Nucleotide Polymorphisms (SNPs) genotyping section: sample dish QC ≥ 0.82 and QC call rate ≥ 66.

Regarding SNP quality control (QC), pruning was performed to retain sites with low missing genotype rates (< 20%), low minimum allele frequency (MAF) ≥ 0.01, and loci under non-linkage disequilibrium (indep-pairwise 50 10 0.3). Additionally, a separate sample quality check was conducted to remove samples with high missing (> 5%). This is now detailed in the Methods section, under **Single Nucleotide Polymorphisms (285-290) and for quality control and validation kindly refer to line 228-236 of the clean copy**

Line 147- 148. The author should describe clarify the call rate data and present data analysis on an additional file to allow data replication and reproducibility.

Call rate was considered when assessing sample quality prior to genotyping. Please refer to the Single Nucleotide Polymorphisms (SNPs) genotyping subsection. Also, validity and replicability of the SNP chip has been assessed in a recent publication by Gomez-Palacio et al. 2024. We point to this paper in the manuscript now in line (155-157).

Line 150-.151 the Mendelian expectations based on SNPs screening, please clarify.

Concerns has been addressed kindly refer to line 165-185 of the clean copy. More details can be found in Evans et al. 2015.

Line 155. Should include data quality check, this full section is missing.

Concerns has been addressed above. Kindly refer to line 228-2360 of the clean copy

Line 160-170: The sample genotype calls rates are produced by the Axiom algorithm, but the results are not been presented on an additional file. The population stratification was done using PCA, to account for identification of the population structure at sample level. But it could have been better to perform a cross-validation technique in order to assess the robustness of results.

We understand the reviewer's recommendations; however, our manuscript also presents admixture results in two different contexts: one to infer the stratification of our samples among Kenyan samples and another in a global context (Fig 2 and Fig 3). Moreover, a phylogenetic analysis was also included, highlighting the genetic differences among our Kenyan samples (Fig 7).

Line 170-171. At SNPs level, data quality control is described using the PLINK 1.9, to identify and remove conflicting SNPs that deviate significantly from expected patterns. Describe briefly the method used for quality check and validation at SNPs level. This will include, Minor Allele Frequency (MAF), access the SNPs Linkage disequilibrium (LD) and the Hardy-Weinberg equilibrium (HWE).

This concern has been addressed above. Please refer to the Single Nucleotide Polymorphisms (SNPs) genotyping subsection in line 148-175.

Line 178. Only Heterozygosity rate is described for genetic diversity but PIC test procedures if included could have been an asset.

Thank you for the suggestion, however we believe our analysis is sufficient for our goals. PIC test was not done on the analysis.

Results

Line 200: Before presenting Population structure results, the author should start first, data quality check and validation results. An additional file should be attached showing quality check outcome at sample-level, and at the SNPs level.

We have included more information about how quality control was performed at sample-level and SNP-level. We have used the best practices for applying strict filters to obtain a Kenyan dataset with samples and SNPs at high quality. The same parameters were used for different studies in our group to study the genetics of Ae. aegypti in different regions of the world, as Gloria-Soria et al. 2018, Elnour et al. 2022, Kotsakiozi et al., 2018a, Kotsakiozi et al., 2018b.

Additionally, the performance of our *Ae. aegypti* SNP-Chip platform was recently demonstrated (Gómez-Palacios et al., 2024). In this manuscript, populations from Sudan and Sri Lanka were genotyped 3 times to assure its reproducibility. The results showed no statistical difference in genotype reproducibility based on source population. Moreover, our SNP-Chip had also good concordance with WGS at medium depth. Since the current manuscript under review is not about the development of the SNP chip, but focused on the study of Kenya populations, we do not consider relevant for the manuscript to expand on the data quality check and validation. We believe the reviewer had made this request, because in the original version submitted, information on the development of the SNP chip used was omitted. We have now addressed that mistake.

Line 229. Only the population stratification results using PCA are shown, presenting the population structure at sample level. Please, provide evidence of data validation at sample and SNPs level. All genetic Diversity indices should be presented in a table.

In our manuscript, we have used different approaches to assess the genetic structure of our samples as admixture analysis and phylogenetic tests.

Discussion

Discuss first all SNPs validation and genetic diversity Indices before structure and isolation by distance

Thank you for the suggestion. We have now included a section focused on SNP filtering and genetic diversity prior to the beginning of the results and methods.

Recommendations

This paper is addressing the Population genetic analysis of the two devastating subspecies of Aedes aegypti, implicated in the transmission of dengue and chikungunya virus in Kenya. Results generated could account for the understanding of the distribution of epidemics and envisage a better management of the species and the disease control, but:

1. The author should include a clear quality check and validation methods steps, then present related results in the results section.
   We have addressed this concerns above, please refer to responses for Reviewer 1.

2. Section of genetic diversity indices results should be presented in a table form, to preside the structure, PCA and phylogenetic results.

We have addressed this concerns above, please refer to line 305-308 of the clean copy.

The results presented in this paper, although important are insufficient and therefore, make the decision of the paper difficult. The methodology is not rigorous and could not clearly allow the reproducibility of the experiment. Since the results could not be confirmed and validated at the sample and at the SNPs level. This paper; although important, is unfit for publication in Plos Neglected

Click here to access/download
Revised Article with Changes Highlighted
Revised Manuscript with Track Changes.docx
