# Supplementary material for: Population genetic analysis of Aedes aegypti reveals evidence of emerging admixture populations in coastal Kenya
Source: PLoS Negl Trop Dis. 2025 May 20;19(5):e0013041. doi: 10.1371/journal.pntd.0013041 (PMC12140423; doi:10.1371/journal.pntd.0013041)
Supplement: Attachment — Submitted filename: Response to Reviewers_Francis.docx [file pntd.0013041.s007.docx]

Francis Mulwa Musili

KEMRI CVR

TO

THE EDITORS

PLOS NTD

RE: **Response to comments Raised by reviewers on the Manuscript**

**Minor corrections**

**Line 150:** How did the author screened SNPs using chip, in order to meet the Mendelian expectations.

Different concepts must be considered when designing an SNP chip, such as including only single-copy markers in the genome, ensuring they are inherited in a Mendelian fashion, and selecting markers associated through linkage disequilibrium due to evolutionary processes rather than physical proximity. All these concepts were considered by our group during the design of *Ae. aegypti* Axiom_aegypti1 platform and are detailed in Evans et al. (2015). As it is described in this paper, Mendelian inherence was studied by genotyping cohorts of F1 offspring and their respective parents, and the results showed that our custom platform contains probes for > 25,000 well-validated biallelic SNPs. In our current work we have eliminated those SNPs that Evans et al. ([2015](https://onlinelibrary.wiley.com/doi/10.1111/mec.15590#mec15590-bib-0023)) found not to follow Mendelian inheritance patterns. We thank the reviewer for bringing this point to our attention. A section describing the SNP chip was missing from the original submission due to an oversight and has now been added starting lines 271, under a new section labeled “**Single Nucleotide Polymorphisms (SNPs) genotyping,** which provides more details on the genotyping platform. **Kindly refer to line 148-175 of the clean copy**

**Line 152:** the author should avoid vague expression such as …to eliminate SNPs .which did not genotype well, instead ,use technical and appropriate expressions such as … to eliminate missing data at those specific locations on the genome .Or simply state that … to eliminate missing data which did not confidently allow to identify the genetic variation at those SNP sites.

**Well noted the sentence has been rephrased, kindly refer to line 171-173 of the clean copy**

**The statistical data analysis methods section is missing in this paper**

We thank the reviewer for pointing out it. We have added more detailed information on the correlation test (lines 285-290.) and additional file for the pairwise test provided.

**Major corrections**

**Line 109. Methods. The author should include a section on data quality control/check**

We have added a citation describing the aegypti SNP chip development (Evans et al. 2015). Futhermore, the manuscript now includes additional information regarding the removal of samples that do not meet the quality requirements as part of the Single Nucleotide Polymorphisms (SNPs) genotyping section: sample dish QC ≥ 0.82 and QC call rate ≥ 66.

Regarding SNP quality control (QC), pruning was performed to retain sites with low missing genotype rates (< 20%), low minimum allele frequency (MAF) ≥ 0.01, and loci under non-linkage disequilibrium (indep-pairwise 50 10 0.3). Additionally, a separate sample quality check was conducted to remove samples with high missing (> 5%). This is now detailed in the Methods section, under **Single Nucleotide Polymorphisms (285-290) and for quality control and validation kindly refer to line 228-236 of the clean copy**

**Line 147- 148. The author should describe clarify the call rate data and present data analysis on an additional file to allow data replication and reproducibility.**

Call rate was considered when assessing sample quality prior to genotyping. Please refer to the Single Nucleotide Polymorphisms (SNPs) genotyping subsection. Also, validity and replicability of the SNP chip has been assessed in a recent publication by Gomez-Palacio et al. 2024. We point to this paper in the manuscript now in line (155-157).

**Line 150-.151 the Mendelian expectations based on SNPs screening, please clarify.**

Concerns has been addressed kindly refer to line 165-185 of the clean copy. More details can be found in Evans et al. 2015.

Line 155. Should include data quality check, this full section is missing.

Concerns has been addressed above. Kindly refer to line 228-2360 of the clean copy

**Line 160-170:** The sample genotype calls rates are produced by the Axiom algorithm, but the results are not been presented on an additional file. The population stratification was done using PCA, to account for identification of the population structure at sample level. But it could have been better to perform a cross-validation technique in order to assess the robustness of results.

We understand the reviewer's recommendations; however, our manuscript also presents admixture results in two different contexts: one to infer the stratification of our samples among Kenyan samples and another in a global context (Fig 2 and Fig 3). Moreover, a phylogenetic analysis was also included, highlighting the genetic differences among our Kenyan samples (Fig 7).

**Line 170-171.** At SNPs level, data quality control is described using the PLINK 1.9, to identify and remove conflicting SNPs that deviate significantly from expected patterns. Describe briefly the method used for quality check and validation at SNPs level. This will include, Minor Allele Frequency (MAF), access the SNPs Linkage disequilibrium (LD) and the Hardy-Weinberg equilibrium (HWE).

This concern has been addressed above. Please refer to the Single Nucleotide Polymorphisms (SNPs) genotyping subsection in line 148-175.

**Line 178.** Only Heterozygosity rate is described for genetic diversity but PIC test procedures if included could have been an asset.

Thank you for the suggestion, however we believe our analysis is sufficient for our goals. PIC test was not done on the analysis.

**Results**

**Line 200:** Before presenting Population structure results, the author should start first, data quality check and validation results. An additional file should be attached showing quality check outcome at sample-level, and at the SNPs level.

We have included more information about how quality control was performed at sample-level and SNP-level. We have used the best practices for applying strict filters to obtain a Kenyan dataset with samples and SNPs at high quality. The same parameters were used for different studies in our group to study the genetics of Ae. aegypti in different regions of the world, as Gloria-Soria et al. 2018, Elnour et al. 2022, Kotsakiozi et al., 2018a, Kotsakiozi et al., 2018b.

Additionally, the performance of our *Ae. aegypti* SNP-Chip platform was recently demonstrated (Gómez-Palacios et al., 2024). In this manuscript, populations from Sudan and Sri Lanka were genotyped 3 times to assure its reproducibility. The results showed no statistical difference in genotype reproducibility based on source population. Moreover, our SNP-Chip had also good concordance with WGS at medium depth. Since the current manuscript under review is not about the development of the SNP chip, but focused on the study of Kenya populations, we do not consider relevant for the manuscript to expand on the data quality check and validation. We believe the reviewer had made this request, because in the original version submitted, information on the development of the SNP chip used was omitted. We have now addressed that mistake.

**Line 229.** Only the population stratification results using PCA are shown, presenting the population structure at sample level. Please, provide evidence of data validation at sample and SNPs level. All genetic Diversity indices should be presented in a table.

In our manuscript, we have used different approaches to assess the genetic structure of our samples as admixture analysis and phylogenetic tests.

**Discussion**

Discuss first all SNPs validation and genetic diversity Indices before structure and isolation by distance

Thank you for the suggestion. We have now included a section focused on SNP filtering and genetic diversity prior to the beginning of the results and methods.

**Recommendations**

This paper is addressing the Population genetic analysis of the two devastating subspecies of Aedes aegypti, implicated in the transmission of dengue and chikungunya virus in Kenya. Results generated could account for the understanding of the distribution of epidemics and envisage a better management of the species and the disease control, but:

1. The author should include a clear quality check and validation methods steps, then present related results in the results section.

We have addressed this concerns above, please refer to responses for Reviewer 1.

2. Section of genetic diversity indices results should be presented in a table form, to preside the structure, PCA and phylogenetic results.

We have addressed this concerns above, please refer to line 305-308 of the clean copy.

The results presented in this paper, although important are insufficient and therefore, make the decision of the paper difficult. The methodology is not rigorous and could not clearly allow the reproducibility of the experiment. Since the results could not be confirmed and validated at the sample and at the SNPs level. This paper; although important, is unfit for publication in Plos Neglected
